# Assay-ready Cryopreserved Cell Monolayers Enabled by Macromolecular Cryoprotectants

**DOI:** 10.1021/acs.biomac.2c00791

**Published:** 2022-08-16

**Authors:** Ruben M. F. Tomás, Akalabya Bissoyi, Thomas R. Congdon, Matthew I. Gibson

**Affiliations:** † Department of Chemistry, University of Warwick, Gibbet Hill Road, Coventry CV4 7AL, U.K.; ‡ Division of Biomedical Sciences, Warwick Medical School, University of Warwick, Gibbet Hill Road, Coventry CV4 7AL, U.K.; § Cryologyx Ltd., 71-75 Shelton Street, London WC2H 9JQ, U.K.

## Abstract

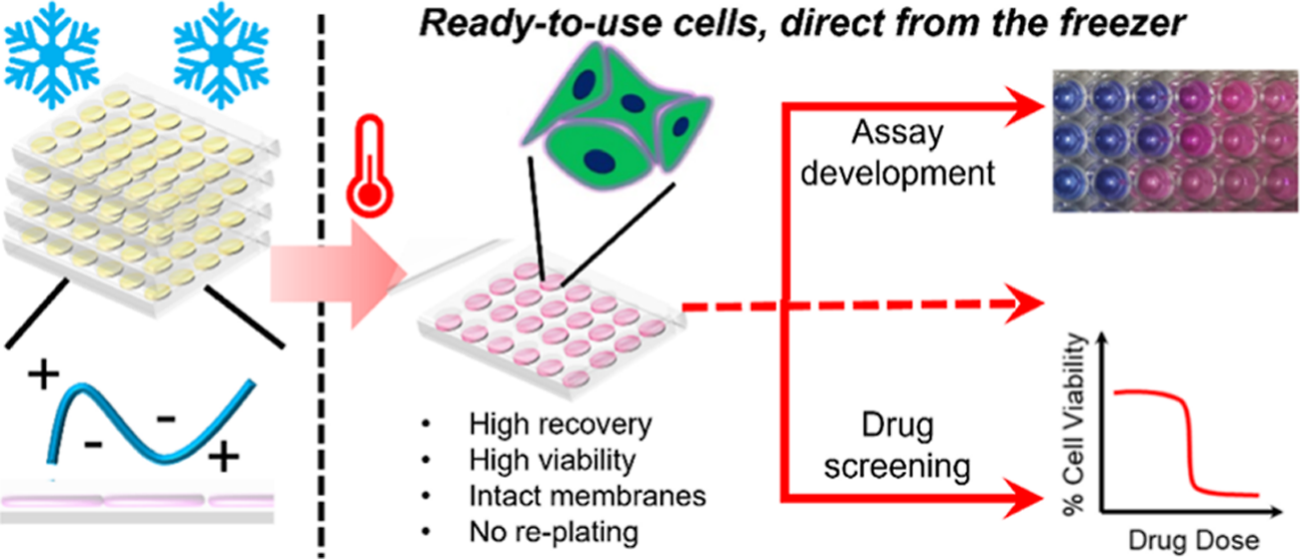

Cell monolayers underpin the discovery and screening of new drugs and allow for fundamental studies of cell biology and disease. However, current cryopreservation technologies do not allow cells to be stored frozen while attached to tissue culture plastic. Hence, cells must be thawed from suspension, cultured for several days or weeks, and finally transferred into multiwell plates for the desired application. This inefficient process consumes significant time handling cells, rather than conducting biomedical research or other value-adding activities. Here, we demonstrate that a synthetic macromolecular cryoprotectant enables the routine, reproducible, and robust cryopreservation of biomedically important cell monolayers, within industry-standard tissue culture multiwell plates. The cells are simply thawed with media and placed in an incubator ready to use within 24 h. Post-thaw cell recovery values were >80% across three cell lines with low well-to-well variance. The cryopreserved cells retained healthy morphology, membrane integrity, proliferative capacity, and metabolic activity; showed marginal increases in apoptotic cells; and responded well to a toxicological challenge using doxorubicin. These discoveries confirm that the cells are “assay-ready” 24 h after thaw. Overall, we show that macromolecular cryoprotectants can address a long-standing cryobiological challenge and offers the potential to transform routine cell culture for biomedical discovery.

## Introduction

Convenient, ready-to-use from the freezer, cell-based assays could accelerate and simplify the discovery of pharmaceutically active compounds, biocompatibility testing, assay development, and discovery of cell signaling and disease pathways. Cryopreservation enables the long-term storage of cells to prevent phenotypic drift associated with continuous culture and allows the distribution of standardized cells around the globe. However, the cold stress of cryopreservation can lead to cell death as described by Mazur’s two-factor hypothesis.^1^ This includes excessive dehydration by slow freezing, due to excessive exposure to high salt concentrations from residual unfrozen water, and intracellular ice formation (IIF) (and recrystallization during slow warming) during rapid freezing. To mitigate the damage caused during cryopreservation, a cryoprotective agent (CPA) is essential, with DMSO (dimethyl sulfoxide) being the most widely used cryoprotectant for nucleated mammalian cells.^2^ During typical slow-freezing cryopreservation (1 °C min^–1^), as cellular dehydration occurs, DMSO replaces lost water to reduce cell death from excessive dehydration and IIF.^3,4^ DMSO also minimizes osmotic shock by reducing electrolyte concentration in residual unfrozen solution during freezing.^5^

Mammalian cells are typically cryopreserved in suspension, which often gives high yields,^6−8^ however for most laboratory-based applications (such as toxicity or uptake screening) cells are used as adherent monolayers, thus, creating a mismatch between the format used for storage and that used to generate data. From suspension, cryopreserved cells must be thawed, seeded, and propagated through several flasks until enough cells can be plated into well plates for the desired application. Hence, a cell biologist is required to devote a large portion of their time to cell preparation, approximately 1–3 weeks depending on the cell proliferation rates. In essence, more time is spent “handling” the cells rather than conducting research or “value-adding” activities with the cells. The routine handling of cells is also plagued with significant single-use plastic waste,^9^ which presents environmental concerns. Furthermore, exploring different cell lines (e.g., to look for a toxic response or drug delivery) can require time, financial resources, and extensive cell culture knowledge to establish, such as optimum confluency for activity and substrate specificity.^10−13^

A (deceptively) simple solution to the abovementioned challenges would be to cryopreserve cells directly in microwell plate substrates. In an ideal world, cells could be removed from cold storage in an “assay-ready” format, thawed, and used 24 h post-thaw, removing weeks of laboratory researcher time, facilitating high-throughput screening, and ensuring phenotypically identical cells. Furthermore, highly desirable characteristics of adherent cells could be preserved, including neuron networks and tight cellular junctions.^14,15^ However, DMSO is unable to cryopreserve cells in the monolayer format, with typically ≤30% of cells recovered after freeze/thaw and the presence of cell debris, which could compromise assay results.^16^ IIF is a particular problem for the cryopreservation of cells as monolayers (and spheroids), compared to suspension cells, as cell–cell contacts promote the propagation of intracellular ice,^17−19^ which is usually fatal and contributes to the low cell recovery rates (although IIF is not always deleterious).^20^ To increase post-thaw cell recovery, IIF can be reduced by directional freezing^21^ or manually inducing nucleation at warmer temperatures, for example using N_2_ ice mist,^22^ or pollen extracts^23^ however reproducibly inducing nucleation is difficult using manual methods and chemically defined nucleating agents are not available.

To address the challenges of cryopreservation, natural^7,24−27^ and synthetic^28−31^ small molecules and macromolecules have been explored, which are inspired by extremophiles^32^ and capable of inhibiting ice recrystallization (IRI) and modulating ice growth and formation.^33^ IRI has been shown to benefit red blood cell cryopreservation;^28,30,34,35^ however, the magnitude of benefit during monolayer or suspension cryopreservation of nucleated cells is limited.^36−38^ Protective osmolytes such as trehalose and l-proline can mitigate some damage during monolayer freezing,^36^ increasing the recovery of Neuro-2a cell monolayers from 13 to 53%^39^ and HepG2 cells from 13 to 42%.^40^ Matsumura and Hyon reported the cryoprotective properties of polyampholytes, polymers bearing mixed anionic/cationic side chains, using carboxylated poly(ε-lysine) (PLL) and 10% (v/v) DMSO, which performed twice as well as DMSO alone for freezing of L929 and rat bone marrow mesenchymal stem cells as monolayers.^41^ The mechanism of protection of polyampholytes is not yet clear, but there is evidence that salts are trapped by a matrix surrounding the cells to minimize osmotic damage, while ensuring sufficient dehydration to prevent spontaneous IIF.^42,43^ Polyampholytes may also interact/protect the cell membrane,^44^ similar to how antifreeze proteins can protect liposome models.^45^ COOH-PLL has also been used to preserve oocytes,^46^ chondrocyte sheets,^47^ and human stem mesenchymal stem cells.^48,49^ However, in these systems, vitrification is often required to achieve high cell recovery, necessitating significant volumes of organic solvent cryoprotectants, such as 6.5 M ethylene glycol.^48^ The broad applicability of the ampholyte scaffold has been shown by deploying backbones with mixed charged side-chains, with reports suggesting that either excess anionic^48,50^ or 1:1 anionic/cationic groups yield the strongest cryoprotective activity.^51−53^ Bailey et al. introduced a synthetically scalable polyampholyte based on the ring open polymerization poly(methyl vinyl ether-*alt*-maleic anhydride).^54^ The use of a maleic anhydride-containing polymer ensures a 1:1 ratio of cationic to anionic groups, unlike heterogeneous materials based on random copolymerization^52^ or carboxylation of PLL.^41^ This polyampholyte enhanced the post-thaw recovery of a panel of cell lines (A549, MC-3T3, and Neuro-2a cells) compared to DMSO alone and the suspension cryopreservation of stem cells.^54,55^ Despite these advances, there is still no demonstration that cells frozen as monolayers are “assay ready”, whereby they can be removed from the freezer and, with minimal user intervention, be ready to use. To achieve this, an in-depth analysis of cellular health and function is required and the cell lines selected should be of crucial importance to toxicological or screening assays (such as liver cells) to have a significant impact.^56,57^

Herein, we demonstrate that the polyampholyte derived from poly(methyl vinyl ether-*alt*-maleic anhydride) and dimethylamino ethanol enables near-quantitative recovery of intact and viable cell monolayers 24 h following removal from a −80 °C freezer, with minimal processing and handling. High cell recovery was demonstrated for adherent cell lines that are widely used in the drug discovery field including A549, HepG2, and Caco-2 cells. Banked “assay-ready” cells were frozen at multiple cell densities, designed for adaptability toward desired applications, and could be stored for over 1 month in a conventional −80 °C freezer. A large panel of post-thaw proliferation, metabolic, membrane integrity, apoptosis, cell cycle, and morphological analyses confirmed that cell monolayers are healthy, function as normal, and are “assay ready”. Finally, a model drug screening test was undertaken using doxorubicin, a chemotherapeutic drug. Our work demonstrates that adherent cells can be cryopreserved so that they are “assay ready” direct from the freezer with minimal handling, facilitating the drug discovery process and reducing the significant time burden associated with routine cell culture.

## Experimental Section

### Materials

Poly(methyl vinyl ether-*alt*-maleic anhydride) (*M*_n_ ≈ 80 kDa), tetrahydrofuran (THF), dimethylamino ethanol, deuterium oxide (D_2_O), type I solution from rat tail, doxorubicin hydrochloride (98%), Eagle’s minimum essential media (MEM), fetal bovine serum (FBS) (non-USA origin), MEM nonessential amino acid (NEAA) solution (100×), 0.4% Trypan blue, dimethyl sulfoxide Hybri-Max, and RNase A from bovine pancreas were purchased from Merck (Gillingham, UK). Dulbecco phosphate-buffered saline (DPBS), Ham’s F-12K (Kaighn’s) medium (F-12K), Antibiotic–Antimycotic (100×) (PSA), Trypsin–EDTA (0.25%), CellEvent Caspase-3/7 Detection Reagent, Live/Dead Viability/Cytotoxicity Kit for mammalian cells, phenol-free DMEM/F-12, and propidium iodide were purchased from Fisher Scientific (Loughborough, UK). Resazurin tablets were purchased from Scientific Laboratory Supplies (Nottingham, UK). P450-Glo CYP3A4 Assay and P450-Glo CYP2C9 Assay were purchased from Promega (Wisconsin, USA).

### Physical and Analytical Methods

#### Nuclear Magnetic Resonance Spectroscopy

^1^H and ^13^C NMR spectra were recorded on a Bruker HD-400 spectrometer, operating at 293 K using deuterated solvents purchased from Sigma-Aldrich. Chemical shifts were reported as δ in parts per million (ppm) relative to residual nondeuterated solvent resonances (D_2_O ^1^H: δ = 4.71 ppm). Bruker Topspin 3.5 Software was used to process and export spectra.

#### Infrared Spectroscopy

FTIR spectra were acquired using an Agilent Cary 630 FTIR (Agilent Technologies, Connecticut, USA) spectrometer equipped with a single reflection diamond ATR accessory with a 45° angle of incidence, a 1 mm diameter sampling surface (200 μm active area), and a rotating pressure clamp (applying maximum pressure). Scans (128) were obtained of dried, crushed samples between 4000 and 650 cm^–1^ with a spectral resolution <2 cm^–1^, wavenumber accuracy of 0.05 cm^–1^, and wavenumber reproducibility of 0.005 cm^–1^. Gain, aperture, scan speed, and filter were all set to auto. Agilent MicroLab Software, version B.05.4, was used to process and export spectra.

#### Microscopy

An Olympus CX41 microscope equipped with a UIS-2 20×/0.45/∞/0–2/FN22 lens and blue and green excitation lasers were used for imaging. Image processing was completed using ImageJ (v1.52).

#### Flow Cytometry

A BD Accuri C6, equipped with 488 (solid) and 640 (diode) nm lasers and 533/30, 585/40, and 670 LP filters, was used for flow cytometry experiments. Instrument calibration was completed using BD Biosciences CS&T beads. Maintenance was completed with BD Biosciences cleaning solutions. All samples were analyzed with a flow rate of 14 μL min^–1^ (10 μm core) and thresholding maintained at 10,000. BD CSampler Plus software (v 1.0.34.1) was used for data collection and processing. FlowJo X 10.0.7r2 (Tree Star, Ashland, USA) was used for all statistical analysis and plotting of flow cytometry data.

#### Statistical Analysis

Data were plotted and analyzed using Graphpad Prism 9 with a one-way analysis of variance (ANOVA) followed by a comparison of experimental groups with the appropriate control group (Tukey’s post hoc test).

#### Cryomicroscopy

Intracellular ice growth was assessed as described previouslys^58^ Cells were seeded on a 14 mm round glass coverslip at a density of 1 × 10^6^ cells mL^–1^. The coverslip of adhered cells was placed on a quartz crucible containing cryoprotectant solution (5 μL), consisting of polyampholyte (40 mg mL^–1^) and 10% DMSO, which was subsequently placed on a Linkam BCS 196 cryostage (Linkam Scientific Instruments, Salford, UK). Cells were incubated at 20 °C for 10 min, to achieve equilibrium, frozen at −40 °C at 1–5 °C min^–1^, and warmed to RT at 20 °C/min. Lynksis 32 software was used to control the cryostage parameters. All images were captured using an Olympus CX41 microscope equipped with a UIS-2 20×/0.45/∞/0–2/FN22 lens (Olympus Ltd.) and a Canon EOS 500D SLR digital camera. Image processing was completed using ImageJ (v1.52).

#### Osmolarity Measurement

An Osmomat 3000 (Gonotec, Berlin, Germany) freezing point depression osmometer was used to test osmolality. The two-point instrument calibration was completed with distilled water, possessing an osmolality of 300 mOsmol kg^–1^, and sodium chloride solution, with an osmolality of 2000 mOsmol kg^–1^. Solutions containing cell culture base media, 10% FBS, 10% DMSO, and/or 40 mg mL^–1^ of polyampholyte were subsequently measured.

### Cryoprotective Polyampholyte Synthesis

The polyampholyte was synthesized as described by Bailey et al., at a larger scale.^54^ Poly(methyl vinyl ether-*alt*-maleic anhydride) with an average *M*_n_ ≈ 80 kDa (10 g) was stirred in THF (100 mL) and heated to 50 °C until dissolved. After dissolution, dimethylamino ethanol (∼10 g) was added in excess, with the mixture turning from clear colorless to a pink waxy solid. Following 30 min, the waxy solid was dissolved in water (100 mL) and left to stir overnight. The remaining THF was removed under vacuum, and the resulting solution was purified in dialysis tubing (Spectra/Por, 12–14 kDa MWCO) for 72 h with 6 water changes. The resulting solution was freeze-dried to form a white solid, which was analyzed using ^1^H and ^13^C NMR (in D_2_O) and IR spectroscopy. Polyampholyte solutions in D_2_O were analyzed after 3 months to determine potential degradation routes.

^1^H NMR (300 MHz, D_2_O) δ_ppm_: 4.71 ppm (D_2_O solvent peak, s), 4.38 ppm (C**H**_**2**_–O–CH_3_, br s, 2H), 3.81 ppm (COO–C**H**_**2**_, br s, 2H), 3.67 ppm (dioxane internal standard peak, s), 3.39 ppm (backbone C**H**–C**H**, br s), 3.18 ppm (OCH_2_–C**H**_**2**_, t), 2.86 (O–C**H**_**3**_, s), 2.82 ppm (N–(C**H**_**3**_)_2_, s), 1.77 ppm (backbone C**H**_**2**_–CH, br s, 2H), 0.78 ppm (end group C**H**_**3**_’s, t). ^13^C NMR (300 MHz, D_2_O) δ_ppm_: 66.6 ppm (dioxane internal standard peak), 58.8 ppm (HCOO–**C**H_2_), 55.3 ppm (**C**H_2_–N(CH_3_)_2_), 43.1 ppm (**C**H–COOH), 42.8 ppm (**C**H_3_)_2_–N. IR ν cm^–1^: 3650–2940 cm^–1^ (br w, O–H acid stretch), 1724 cm^–1^ (s, C=O stretch), 1560 cm^–1^ (w, O=C–O– carboxylate), 1342 cm^–1^ (w, C–N stretch), 1225 cm^–1^ (w, C–O stretch).

### Cell Culture

Human Caucasian lung carcinoma cells (A549, ATCC) were cultured in Ham’s F-12K (Kaighn’s) medium (F-12K) supplemented with 10% FBS and 100 units mL^–1^ penicillin, 100 μg mL^–1^ streptomycin, and 250 ng mL^–1^ amphotericin B (1% PSA). Human liver hepatocellular carcinoma cells (HepG2, ECACC 85011430) were cultured in Eagle’s MEM supplemented with 10% FBS, 1% NEAA, and 1% PSA. Human Caucasian colon adenocarcinoma cells (Caco-2, ECACC 86010202) were cultured in MEM media supplemented with 20% FBS and 1% PSA. Cells were incubated at 37 °C and 5% CO_2_ and passaged every 3–4 days, before reaching 70–80% confluency. Cells were dissociated using a balanced salt solution containing trypsin (0.25%) and EDTA (1 mM).

### Cryopreservation of “Assay-ready” Cells in 2-D Monolayers

#### Confluent Monolayer Freezing

A549 (300k/well), HepG2 (400k/well), and Caco-2 (150k/well) cells were seeded on 24 well plates (Greiner Bio-one, 662160). HepG2 and Caco-2 cells were seeded onto collagen (Type I solution from rat tail Merck, C3867) coated plates. Cells were incubated to allow attachment to the substrate (24 h for A549 and HepG2, 48 h for Caco-2). Cell media was removed, and cells were incubated at RT with 40 mg mL^–1^ of polyampholyte dissolved in cell media (either F12-K or MEM), 10% DMSO, and 10% FBS for 10 min. Polyampholyte solutions were subsequently removed, and the 24 well plates were positioned on a Corning XT CoolSink 96F and placed in a −80 °C freezer overnight. The thermoconductive block ensures well-to-well temperature consistency (no “edge effect”), while eliminating the air gap between the bottom of the well plate and the temperature source. As a control, cells were also cryopreserved as monolayers, in the same manner, with 10% DMSO alone. Cells were removed from the −80 °C, immediately thawed with warm complete cell media (37 °C), and placed in the incubator for 24 h. For variable density freezing, the freezing process was described above, however, cells seeded were serially diluted 2-fold across each column (i.e., 6 times along the six columns). Phase contrast images of nonfrozen and frozen cells were taken on an Olympus CX41 microscope equipped with a UIS-2 20×/0.45/∞/0–2/FN22 lens (Olympus Ltd., Southend-on-Sea, U.K.) and a Canon EOS 500D SLR digital camera. Images were processed using ImageJ (v1.52). Following imaging, cells were dissociated with trypsin (0.25%) and EDTA (1 mM), and the suspended cells were stained with trypan blue to count membrane intact cells. Percentage cell recovery was calculated by comparing cell counts immediately before and 24 h after thawing.

### Biochemical Assays

Various assays were completed as a demonstration of perfect cell heath following freeze-thaw, along with immediate assay-readiness status 24 h post-thaw. The previously described cryopreservation method was used for the banking of cells used throughout.

#### Resazurin Cell Viability Assay

A549 (0–35k cells/well) and HepG2 (0–15k cells/well) cells frozen at multiple densities were thawed with warm complete cell media (37 °C). After 24 h, the media was replaced with resazurin solution (500 μL), which was prepared by dissolving 1 tablet (Scientific 300 Laboratory Supplies, CHE3158) in 50 mL of phenol-free DMEM/F-12. Absorbance measurements were obtained at 570 and 600 nm every 30 min/1 h for 4 h to monitor the reduction of resazurin to resorufin by viable cells. Fluorescence measurements were also recorded using a BioTek Synergy HT microplate reader with a 530/25 nm excitation and 590/35 nm emission filter. Nonfrozen cells were also treated with resazurin solution at similar densities to compare against frozen cells. Cells were counted and the exact cell density was plotted against either resazurin reduction (absorbance measurements) or normalized fluorescence intensity, whereby fluorescence measurements were normalized to readings obtained with the highest cell density. Resazurin solutions without any cells were used to provide background measurements.

#### Live/Dead Assay

A549 and HepG2 cells frozen as confluent monolayers were thawed with warm complete cell media (37 °C) and, 24 h post-thaw, were stained with ethidium iodide (2 μM) and calcein (2 μM) in phenol-free DMEM/F-12 (100 μL) for 40 min at RT. Cells were imaged with an Olympus CX41 microscope using phase contrast and blue (calcein) and green (ethidium) excitation lasers. Nonfrozen cells were also stained to provide control images. ImageJ was utilized to count live (calcein) and dead (red) cells to determine the percentage of live, membrane intact, cells.

#### Caspase-3/-7 Real-time Activation Assay

A549 and HepG2 cells frozen as confluent monolayers were thawed with warm complete cell media (37 °C). Following 10 min of incubation, the media was replaced with complete media supplemented with CellEvent Caspase-3/7 Detection Reagent (5 μM). Images were taken at 1, 2, 4, and 24 h time intervals on an Olympus CX41 microscope with a phase contrast channel and a blue excitation laser. Cells were counted using ImageJ, and values were reported as percentage caspase positive cells relative to the total number of cells. Nonfrozen cells were also stained with CellEvent Caspase-3/7 Detection Reagent to provide a baseline reading.

#### CYP Activity

HepG2 cells were frozen at a density of 37.5–150k cells per well, as described above, for 24 h. Cells were thawed with warm complete cell media (37 °C) and incubated for 48 h. To determine innate CYP450 activity, CYP3A4 and CYP2C9 were subsequently measured using the corresponding Promega CYP450 kits. Briefly, media were replaced with a culture medium containing a luminogenic CYP substrate, either CYP3A4/Luciferin-IPA (3 μM, 300 μL, 1 h) or CYP2C9/Luciferin-H (100 μM, 300 μL, 3 h). The CYP substrate was also added to empty wells as a background measurement. The culture medium containing the CYP substrate (25 μL) was transferred to an opaque white 96 well plate, and luciferin detection reagent (25 μL) was added for 20 min at RT. Luminescence was measured on a BioTek Synergy HT microplate reader. The CYP activity of nonfrozen cells was also measured for comparison. Nonfrozen and Frozen HepG2 monolayers were also treated with rifampicin (0–100 μM, 48 h), 24 h post-thaw, to induce further CYP activity and measured as described previously.

### Growth Curve

A549 and HepG2 cells were cryopreserved at a density of 20k and 50k cells/well, respectively, in 24 well plates. Cells were thawed with warm complete cell media (37 °C) and allowed to incubate for 24 h. Cells were counted daily until ∼80–90% confluency was reached, with trypan blue used to stain for membrane intact cells, and proliferation rates were determined.

### Testing Drug Screening Capacity

A549 and HepG2 cells were seeded at the optimum density determined for resazurin assays previously explored (10k and 20k cells per well, respectively), on 24 well plates, taking into consideration proliferation rates and duration of drug screening experiments. Cells were frozen with 40 mg mL^–1^ of polyampholyte, as described above, and thawed with warm complete cell media (37 °C) after 24 h of storage in a −80 °C freezer. Cells were washed with DPBS, 24 h post-thaw, and incubated with doxorubicin (200–0 μg mL^–1^) for a further 24 h. Cells were washed with DPBS, and resazurin solutions (500 μL) were added to the cells. Absorbance measurements were obtained at 570 and 600 nm every 30 min/1 h for 4 h to monitor the reduction of resazurin to resorufin by viable cells. Control cells untreated with doxorubicin were also treated with resazurin solutions to provide a maximum resazurin reduction value of viable cells, and resazurin solutions without any cells were used to provide background measurements.

### Cell Cycle Analysis

A549, HepG2, and Caco-2 cells frozen as confluent monolayers were thawed with warm complete cell media (37 °C) and incubated for 24 h. Cell dissociation was completed using trypsin (0.25%) and EDTA (1 mM) to harvest 1 × 10^6^ cells mL^–1^. Cells were centrifuged and resuspended in cold 70% ethanol (250 μL), added dropwise while vortexing, and fixed for 30 min at 4 °C. Cells were centrifuged at 300*g* for 5 min, and the pellet was washed with DPBS. Centrifugation was performed again, and the resulting pellet was resuspended in a solution of DPBS containing 20 μg/mL PI and 100 μg/mL RNase A for 30 min.^59^ Nonfrozen cells were also stained for comparative cell cycle measurements. Flow cytometry was performed on a BD Accuri C6 equipped with a 488 nm laser and 585/40 filter, and 50,000 events were recorded. BD CSampler Plus software (v 1.0.34.1) was used for data collection and processing. Cell cycle analysis was completed using ModFit LT v4.0.

### Cell Banking and Transportation Capabilities

A549 and HepG2 cells were frozen at a density of 200k cells per well, as described above, and stored for 1 month in a −80 °C freezer or for 5 days in dry ice, to determine the potential for long-term storage and transportation, respectively. Cells were thawed with warm complete cell media (37 °C). After 24 h, cells were dissociated with trypsin (0.25%) and EDTA (1 mM), and the suspended cells were stained with trypan blue to count membrane intact cells. Percentage cell recovery was calculated by comparing cell counts immediately before and 24 h after thawing.

## Results and Discussion

**Figure 1:**
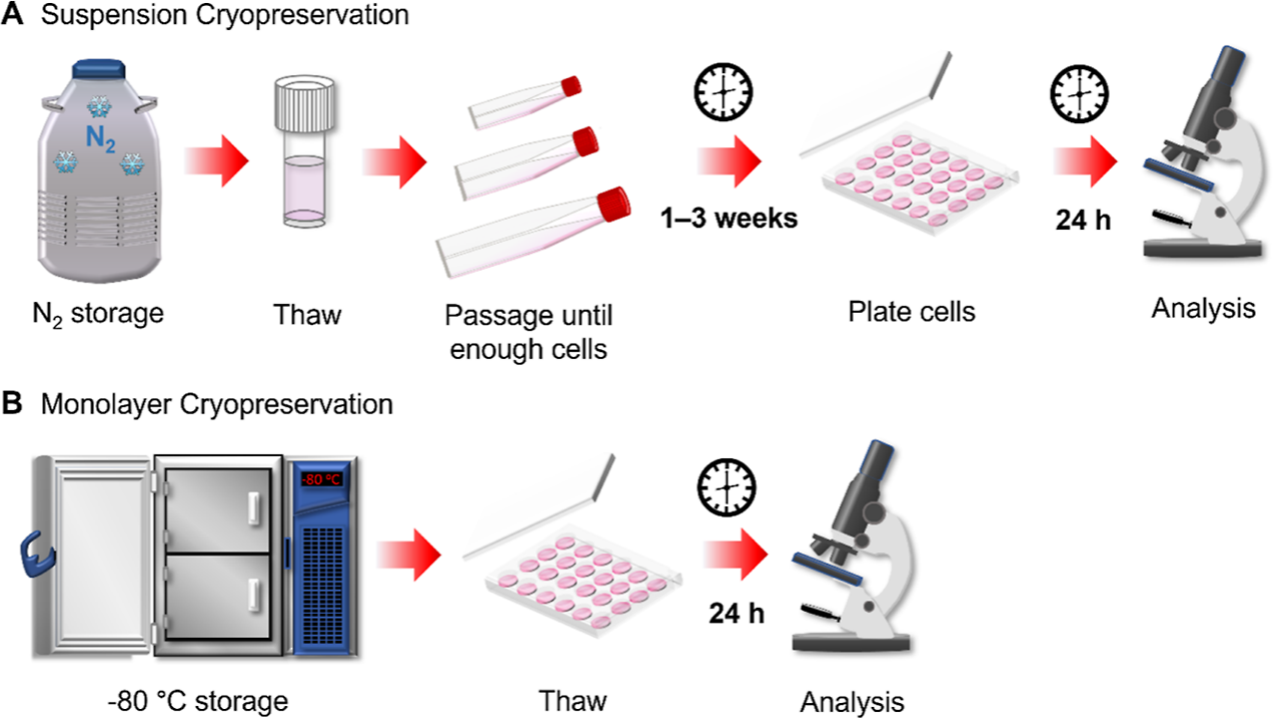
Schematic comparing the processing of cells following cryopreservation in (A) suspension format and (B) an “assay-ready” monolayer format.

**Figure 2:**
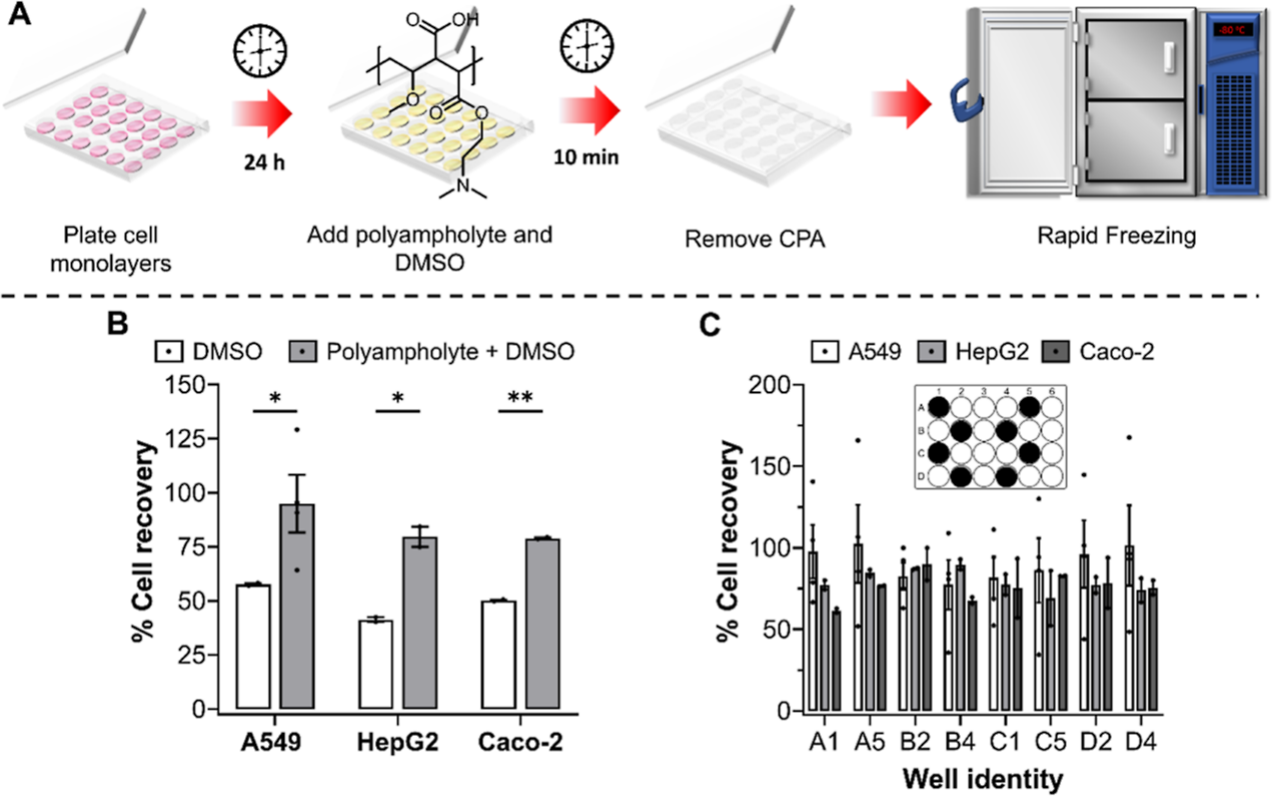
Post-thaw cell recovery of cell monolayers. (A) Schematic of the monolayer cryopreservation process. (B) Percentage cell recovery of A549 (*n* = 4), HepG2 (*n* = 2), and Caco-2 (*n* = 2) cell monolayers following cryopreservation with 10% v/v DMSO (white bar) or 10% v/v DMSO and 40 mg mL^–1^ polyampholyte (grey bar). (C) Percentage cell recovery of A549 (white bar), HepG2 (grey bar), and Caco-2 (dark grey bar) cell monolayers in selected individual wells (filled in black in the 24-well plate schematic) following cryopreservation in 10% v/v DMSO and 40 mg mL^–1^ polyampholyte. Data is presented by average percentage cell recovery ± SEM of *n* independent repeats (ANOVA, Tukey PostHoc; *p ≤ 0.05, **p ≤ 0.01).

Our primary aim is to cryopreserve cells in a format that is ready to use directly from a −80 °C freezer, with no additional processing steps, in particular, to remove the need for routine cell handling, Figure 1. The macromolecular cryoprotectant selected to achieve this was a polyampholyte generated by the ring open polymerization of poly(methyl vinyl ether-*alt*-maleic anhydride) with dimethylamino ethanol, to produce a polymer with 1:1 cationic/anionic groups, which has been previously demonstrated to allow the cryopreservation of A549 monolayers.^54^ The proposed workflow for the cryopreservation and recovery of cell monolayers is displayed in Figure 2A. Briefly, cells seeded in 24 well plates were incubated with CPAs (40 mg mL^–1^ of polyampholyte and 10% (v/v) DMSO) at RT for 10 min. The CPAs were subsequently removed so that only a thin coating remains, and the well plate was placed onto a thermoconductive block in a −80 °C freezer. As part of the process, the excess cryoprotectant is removed before freezing to reduce any potential cytotoxicity from the cryoprotectants, increase freezing rates and facilitate the thawing process. Acker and Mcgann previously confirmed that rapid freezing by removing CPA is beneficial to monolayers, and could promote innocuous (nonfatal) IIF.^60^ Once required for use, the plates were simply removed from the freezer, and cells were rapidly thawed with warm complete cell culture media and placed in an incubator ready to use the following day (24 h). Rapid thawing was completed to minimize the growth of ice crystals in the solid state and cell denaturation due to high electrolyte concentrations.^61^ A post-thaw recovery time of 24 h was selected, as shorter times (4 h) can exaggerate both cell recovery and viability, and programed cell death pathways can take 12–24 h to complete,^62,63^ leading to significant false positive results when discovering innovative cryoprotectants.^6^ For example, a previous polyampholyte was shown to have high immediate post-thaw viability but no viable cells after 24 h.^52^

To evaluate the cryoprotective effect of this polymer, A549 (adenocarcinomic human alveolar basal epithelial cells), HepG2 (human hepatocellular carcinoma), and Caco-2 (human colorectal adenocarcinoma) cells were employed, all of which are widely used as model cell lines. The seeded cells were counted immediately before freezing and 24 h post-thaw to determine the percentage of cells recovered, as shown in Figure 2B. Using our optimized method, remarkably high cell recovery values were obtained for A549 (92.5 ± 4.1%, 4 biological and 20 technical repeats), HepG2 (79.6 ± 8.7%, 2 biological and 22 technical repeats), and Caco-2 (79.3 ± 8.7%, 2 biological and 18 technical repeats) cells, whereas freezing with 10% DMSO alone only achieved an average cell recovery of 49.9% across all cell types, Figure S5. The abnormally high cell recovery values for DMSO alone, compared to previously reported literature values (≤30%),^16^ reflect the optimization of the freezing protocols, which, together with the selection of cryoprotectant, is crucial for the successful cryopreservation of nucleated cells. The minimal handling required to obtain high post-thaw cell recovery values shows the remarkable potency of polyampholyte as a cryoprotectant, transforming sub-useful cell recovery levels to near-quantitative. For comparison, proline preconditioning of A549 cells only achieves a post-thaw cell recovery of ∼50% for the cryopreservation of monolayers^36^ and the post-thaw recovery of HepG2 monolayers cryopreserved in trehalose supplemented solutions is 42%.^40^ Thus, this polyampholyte can protect cell lines that are routinely difficult to cryopreserve for the future banking of cell libraries. As a note, HepG2 and Caco-2 cells required collagen-coated plates to ensure high cell recovery values (Figure S18) by promoting adhesion to the substrate during freezing. To demonstrate reproducibility, two independent researchers were able to freeze six 24 well plates containing cell monolayers with no impact to cell recovery values, Figure S7.

Rather than aggregated cell data across the entire plate, the cell recovery values for a selection of individual wells have also been reported (along with individual repeat values) to exaggerate any variance between wells, Figure 2C. Regardless of the cell type, minimal well-to-well variance was found in the mean cell recovery values, a crucial factor in screening assays where there must be confidence that results aren’t artificially increased or decreased due to variability in cell density across the well plate.

**Figure 3:**
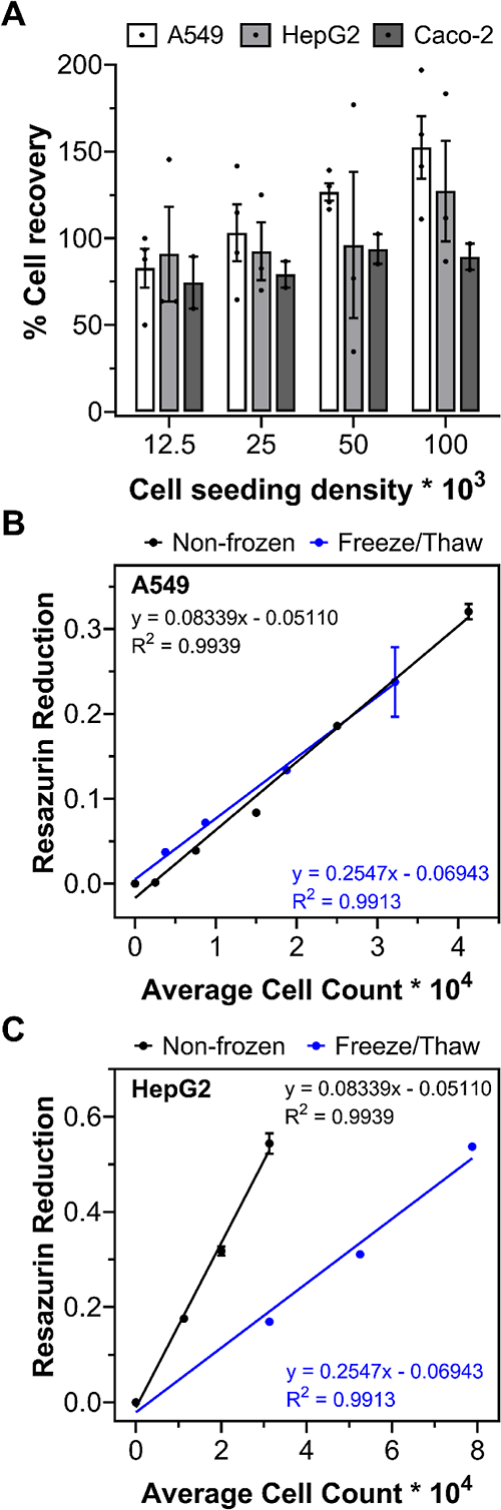
Assay optimization and metabolic activity of cryopreserved cells. (A) Post-thaw percentage cell recovery of A549 (white bar, *n* = 4), HepG2 (grey bar, *n* = 3), and Caco-2 (dark grey bar, *n* = 2) cell monolayers, plated at different seeding densities, following cryopreservation with 10% v/v DMSO and 40 mg mL^–1^ polyampholyte. Data are presented as average % cell recovery ± SEM of *n* independent repeats. A resazurin reduction metabolic assay was completed on frozen (B) A549 and (C) HepG2 plated cells (blue line), 24 h post-thaw, and on nonfrozen, conventionally cultured cells (black). The results are presented as average resazurin reduction ± SEM of 2 independent repeats.

Varying cell seeding densities (12.5k–100k cells per well) also had no influence on the cryopreservation outcome, with over 75% of cells recovered 24 h post-thaw, Figure 3A. Cryopreservation studies focus on confluent cell monolayers, as the general belief is that higher recovery values can be obtained.^60^ However, for cell viability assays, a linear response between cell seeding density and signal output (usually absorbance or fluorescence) is required to ensure that cell viability can be accurately determined. Thus, the ability to cryopreserve cells at multiple cell seeding densities is crucial for assay development and toxicological screening assays. A resazurin reduction metabolic assay was completed on nonfrozen and frozen A549 and HepG2 cells, plated at varying seeding densities, to determine the quantity of cells required to freeze to ensure a linear output with resazurin reduction to resorufin and, thus, allow its use for drug screening, Figure 3B,C, S10, and S11. Both cell lines were able to metabolize resazurin to resorufin 24 h post-thaw and offered a linear response between cell density and absorbance/fluorescence. Although the metabolic activity of A549 cells was undisturbed by the freezing process, HepG2 metabolic activity was halved 24 h post-thaw. The reduction in metabolic activity is expected, halving (or more) of metabolic activity has also been observed for osteoblasts (MG-63),^64^ ovarian tissue,^65^ and mesenchymal stem cells post-thaw.^66^ Despite this, both A549 and HepG2 cells remained functional for use in cell viability assays required for drug screening, see below.

**Figure 4:**
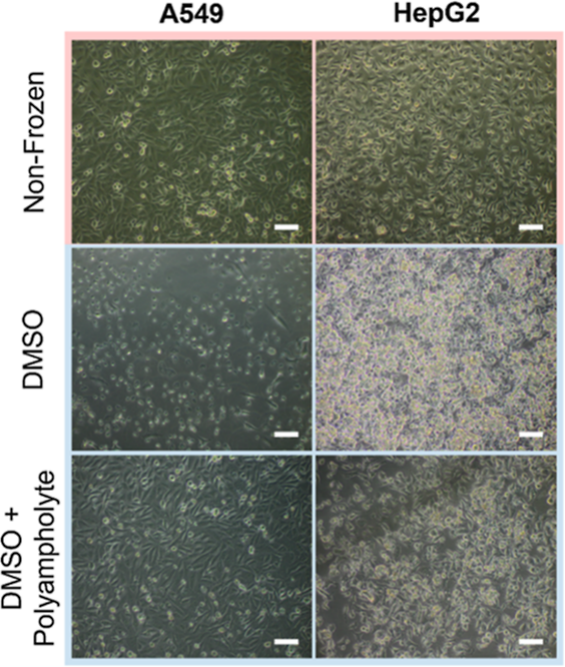
Phase contrast images of A549 and HepG2 monolayers before (nonfrozen) and 24 h after freeze/thaw. Cells were cryopreserved in either 10% v/v DMSO or 10% v/v DMSO and 40 mg mL^–1^ polyampholyte. Scale bar = 100 μm.

Optical microscopy images taken of nonfrozen cells and cells frozen with both polyampholyte and DMSO, shown in Figure 4, illustrate that post-thaw cell morphology is normal and unaffected by the freezing process. A549 and HepG2 cells remained adhered to the well plate, and confluent monolayers were observed. Optical microscopy images were also taken of cells frozen at different cell densities (Figure S8). Again, cells appeared healthy regardless of cell density and were ready for use within the 24 h recovery time provided. Cryopreservation with DMSO alone resulted in spherical cell morphologies associated with unhealthy cells. The injury phenotype has previously been characterized as intracellular freezing damage, indicated by the dark cytosol and disruption of intercellular networks.^21^ Standard rapid freezing methods increase the likelihood of IIF by reducing cell dehydration.^1^ Cryomicroscopy images of A549, HepG2, and Caco-2 cells, Figure S12, confirmed the presence of IIF during freezing (darkening of the cytosol); however, cells preincubated with both polyampholyte and DMSO have a lower surface area compared to DMSO alone (Figures S12 and S13), suggesting that polyampholyte can aid in cellular dehydration as part of their mechanisms of action. Thus, the polyampholyte is not acting to completely remove IIF but to minimize the damage caused by it, so that the intracellular ice formed can be considered innocuous.^60^

**Figure 5:**
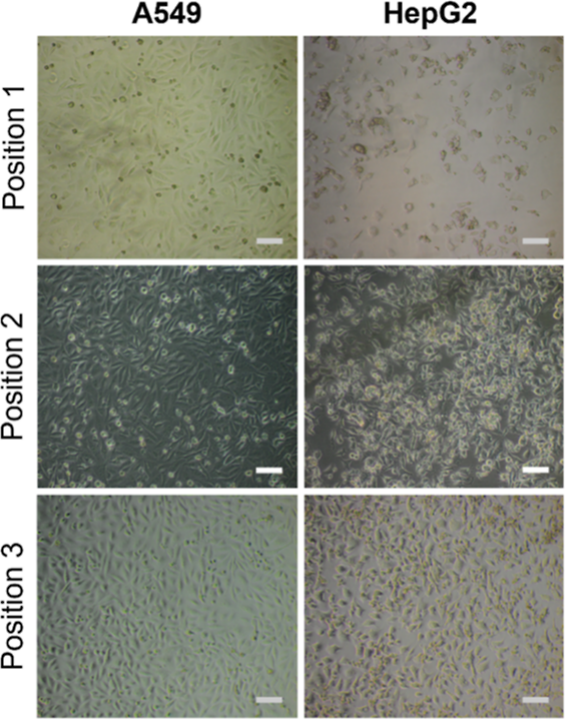
Phase contrast images of A549 and HepG2 monolayers, cryopreserved with 10% v/v DMSO and 40 mg mL^–1^ polyampholyte, were taken at 3 positions within an individual well. Scale bar = 100 μm.

Optical microscopy images were also taken of post-thaw confluent A549 and HepG2 cells from different positions within a single well, to illustrate the low intra-well variance obtained using polyampholyte and DMSO, Figure 5. Cell morphology remained consistently healthy across the different positions. In the case of HepG2, fewer cells were observed in position 1 compared to the other 2 positions. The variation in cell number is likely due to natural variation from the initial seeding process, where traditional seeding methods can result in increased cell aggregation across the circumference and/or the center of wells compared to other positions.^67^ Confluent monolayers were observed in all other positions for both A549 and HepG2 cells.

**Figure 6:**
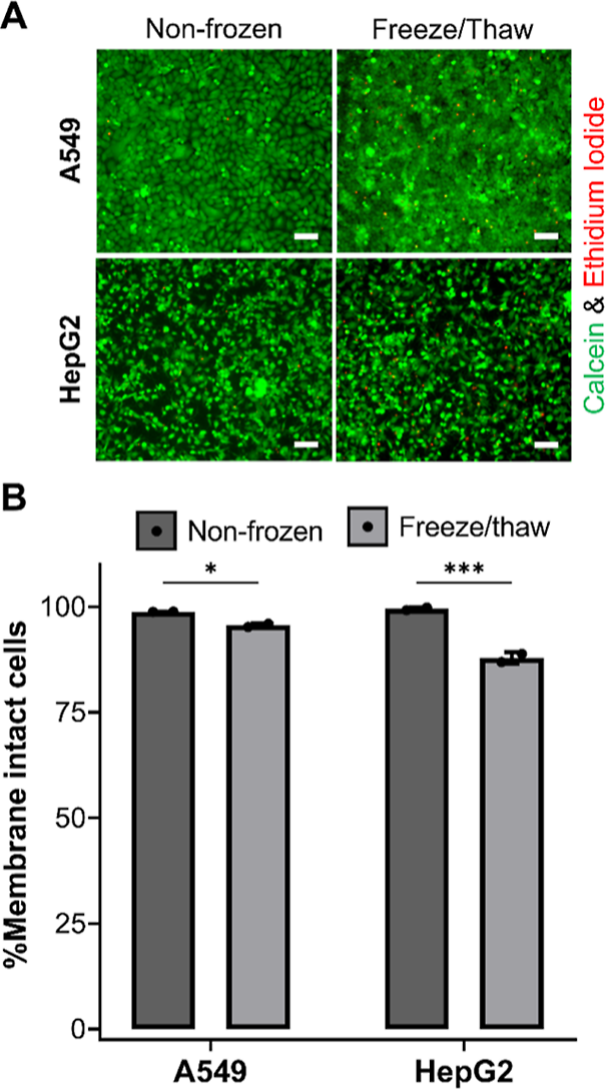
Membrane integrity assessment of cryopreserved cell monolayers. (A) A549 and HepG2 cells were stained with calcein (green, intact membrane) and EI (red, damaged membrane) 24 h following freeze/thaw and imaged. Nonfrozen cells were also stained for comparison. Scale bar = 100 μm. (B) Average percentage of membrane intact (calcein positive) A549 and HepG2 cells were determined before (dark grey bar) and 24 h post-thaw (light grey bar) ± SEM of 2 independent repeats (ANOVA, Tukey PostHoc; *p ≤ 0.05, ***p ≤ 0.001).

To evaluate the quality of the cells post-thaw, a panel of further assays was undertaken. Membrane integrity is a general measure of post-thaw recovery and is a vital assessment for the health of liver cell lines, as enzyme leakage would be detrimental to enzyme activity assays used in hepatotoxicity screening for the identification of bioactive drugs and their potential mechanisms.^68,69^ A549 and HepG2 cells, both nonfrozen and post-thaw, were stained with calcein (green, healthy, intact membrane) and ethidium iodide (EI, red, dead, damaged membrane) to visualize the presence of membrane damage, Figure 6A. Microscopy images immediately revealed few red, EI-positive cells in both nonfrozen and frozen cells, indicating minimal membrane damage. Quantification of the percentage of live cells (i.e., stained positive for calcein), relative to the total number of cells from images, was completed to compare membrane-damaged cells between nonfrozen and frozen cells, Figure 6B. Although a statistically significant decrease in membrane intact cells was observed in cells post-thaw, intact levels remained at ∼96% for A549 cells and ∼88% for HepG2 cells. The absence of membrane damage confirms that physical disruption of cells is avoided during cryopreservation with polyampholyte and DMSO, which reduces the likelihood of necrosis cell death and the risks of introducing bias in any downstream functional assay.

**Figure 7:**
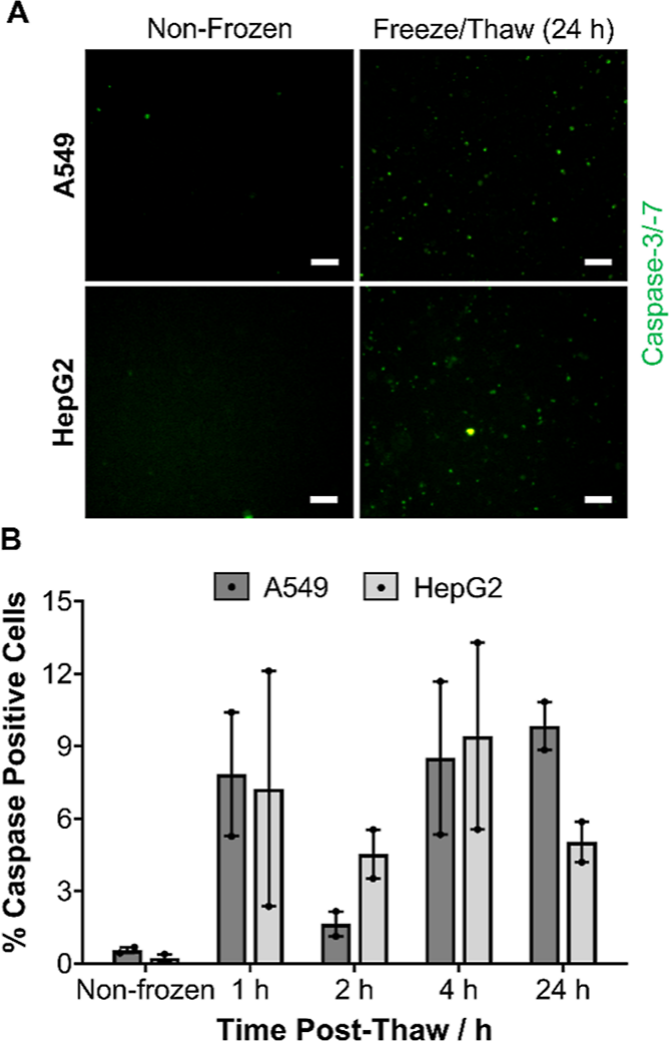
Evaluation of post-thaw apoptosis in cryopreserved monolayers. (A) Sample images of A549 and HepG2 cells stained for executioner caspase-3/-7 activation (green) 24 h post-thaw. Scale bar = 100 μm. Additional real-time images are provided in the Supporting Information. (B) Average percentage of caspase-positive cells were determined at different timepoints post-thaw ± SEM of 2 independent repeats.

Programed cell death (e.g., apoptosis) pathways were also investigated as cryopreservation can activate intrinsic, extrinsic, and calpain apoptosis pathways.^70−72^ Hypothermic solutions and general caspase and ROCK inhibitors (apoptotic inhibitors) have been used to reduce post-thaw apoptotic cells.^70,73,74^ Due to the importance of apoptosis in cryopreservation outcomes, the induction of executioner caspases 3 and 7 (cysteinyl aspartate-specific proteases) in post-thaw confluent A549 and HepG2 cell monolayers was monitored by fluorescence microscopy to visualize the appearance of apoptotic cells over time, Figures 7A and S14. Minimal caspase-3/7 positive cells (green) were observed in microscopy images at all timepoints within the 24 h imaging period. The percentage of caspase-3/7 positive cells relative to the total number of cells present was quantified, Figure 7B, confirming that apoptotic cells were maintained below 10% following freeze/thaw. Apoptotic inhibitors have previously been used to minimize apoptosis following the cryopreservation of mesenchymal stem cells due to the high apoptosis levels (∼40%).^70^ Our system offers cryopreserved monolayer cells with substantially lower apoptosis rates for the cell lines selected, thus the introduction of apoptosis inhibitors would provide minimal benefits. The post-thaw recovery time selected (24 h) is underpinned by these results, to enable apoptotic events to occur and minimize the potential for false-positive results that occur when immediate post-thaw recovery/viability is reported.^6^

**Figure 8:**
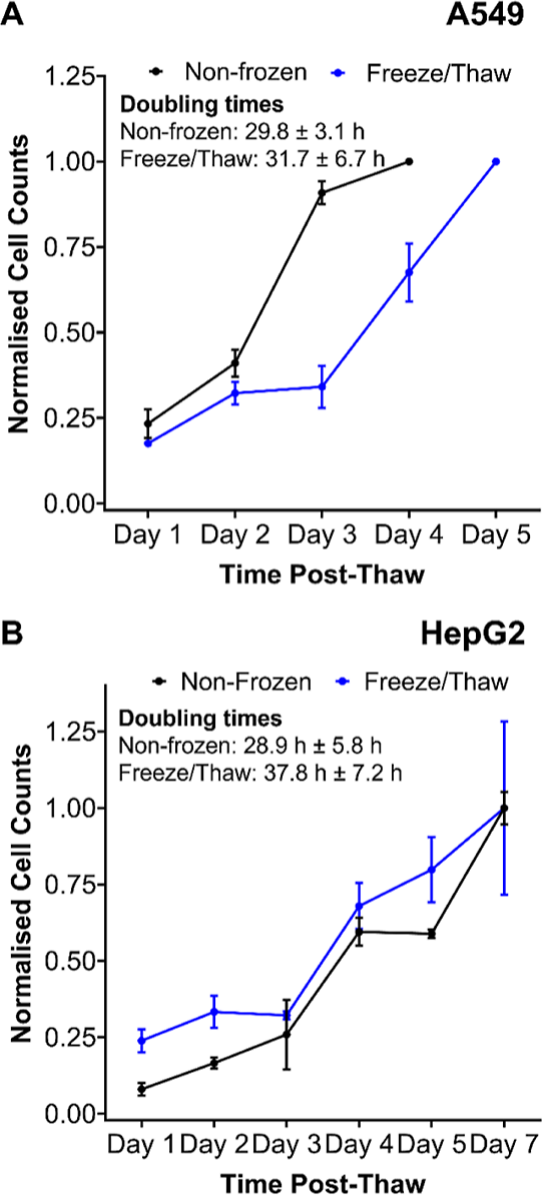
Post-thaw growth curve measurements. Nonfrozen and post-thaw (A) A549 and (B) HepG2 cells were counted daily until fully confluent to determine proliferation rates. Cell counts were normalized relative to the final count and reported ± SEM of 2 independent repeats.

Membrane permeability and apoptosis measurements offer an assessment of immediate cell health post-thaw; however, growth curve measurements are required to determine if cells are healthy long-term. Nonfrozen and post-thaw cells were counted daily, until 80–100% confluency was reached, to determine if the proliferation times of monolayer frozen cells were affected by the freeze/thaw process and have been reported in Figure 8. No significant difference was found between the doubling time of A549 cells before and after freezing; however, HepG2 doubling time increased from 29 to 38 h. Despite this, HepG2 doubling rates of 48 h have been reported, and our lab has also experienced similar doubling rates, so the proliferation rate of post-thaw HepG2 cells is within the normal range expected.^75^

Cryopreservation may significantly alter cell cycle populations up to 48 h post-thaw; however, there are limited reports on this.^76^ Previous cell recovery and viability measurements confirm that, after freezing, cells maintain a “healthy” state, but gaining information into cell cycle population distributions offers additional insight into proliferation capacity,^77^ apoptotic cells,^78^ and cells undergoing repair processes.^79^ Cell cycle population distributions were measured by univariate analysis of cellular DNA following propidium iodide staining, flow cytometry, and deconvolution of the cellular DNA content frequency histograms.^77^ Histograms can be found in the Supporting Information, Figure S15, and the cell cycle populations have been reported in Table 1. Apoptotic cells were not observed in the cell cycle histograms, which are usually present in the sub G0/G1 fluorescence range (due to loss of DNA content). The majority of A549 and Caco-2 cells were either in the S or G2/M phase, a clear distinction from HepG2 cells, which is considered an indicator for increased proliferative potential. Post-thaw, all cell types experienced a decrease in G0/G1 cells and an increase in S-phase cells, a clear indicator that cells are not in G0/G1 arrest (apoptosis indicator)^78,80^ and retain proliferative capacity.^77^ A549 and Caco-2 cells retained similar cell cycle population distributions before and after freezing (24 h post-thaw), whereas clear differences were observed with HepG2 cells which could reflect the changes in proliferation rates between nonfrozen and frozen HepG2 cells previously observed. Regardless, proliferative capacity was retained for all cell types, with minimal indicators of apoptosis found, confirming that cells are functional for use following freeze/thaw.

**Table 1:** Cell Cycle Analysis From Flow Cytometry

	G0/G1 (%)	G2M (%)	S-phase (%)
A549. nonfrozen	28.0	4.6	67.5
A549. post-thaw	21.5	1.5	77.0
HepG2. nonfrozen	68.5	22.4	9.1
HepG2. post-thaw	54.8	12.8	32.4
Caco-2. nonfrozen	43.1	8.0	48.9
Caco-2. post-thaw	39.5	8.0	52.5

**Figure 9:**
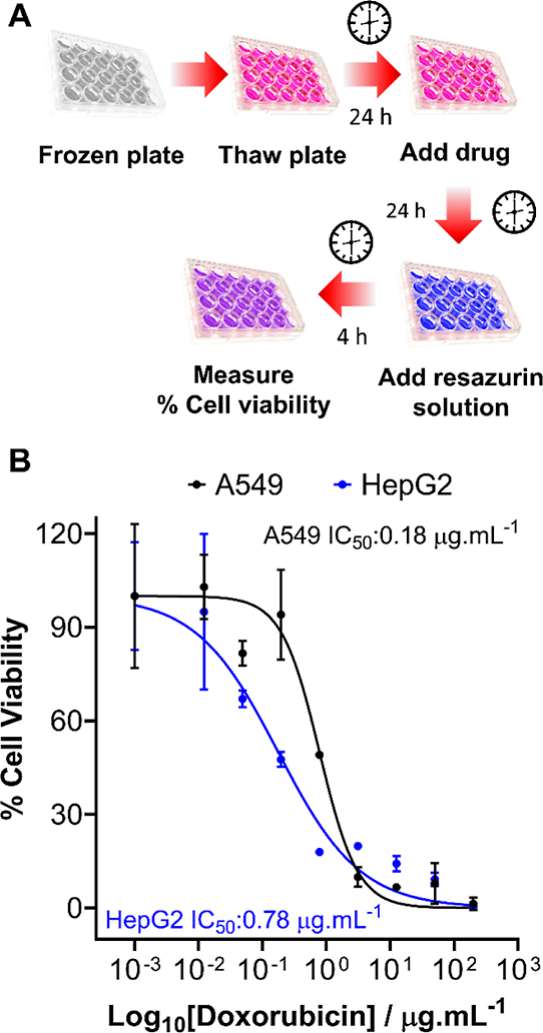
Post-thaw drug screening tests on cryopreserved cell monolayers. (A) Schematic of the drug screening process used on cryopreserved monolayers. (B) Cell viability of A549 (black) and HepG2 (blue) monolayers was monitored by resazurin reduction following treatment with doxorubicin (0–250 μg mL^–1^, 24 h). Percentage cell viability was calculated relative to an untreated control sample ± SEM from 2 independent repeats.

A primary aim of this research program was to provide a scalable method of freezing cells in the monolayer format for use in screening applications, eliminating the need for extensive cell culturing and handing. Obtaining dose–response information, which involves the core principles of pharmacokinetics and pharmacodynamics, is essential for drug screening, to determine the required drug dosage and frequency, as well as its therapeutic index. Thus, a proof-of-concept drug screening experiment was carried out with doxorubicin (a chemotherapeutic) using the previously optimized resazurin reduction cell viability assay. The drug screening workflow is illustrated in Figure 9A. Briefly, cells were cryopreserved, using our system, at the optimal density required to ensure accurate % cell viability calculations. Following 24 h storage, cells were thawed with warm complete cell media and incubated for 24 h, and doxorubicin solutions were added (0–200 μg mL^–1^) for 24 h. Resazurin solutions were subsequently added, and the reduction of resazurin to resorufin was measured at 570 and 600 nm to calculate percentage cell viability and generate dose–response curves, Figure 9B. A sigmoidal relationship was found between log_10_ of doxorubicin concentrations and % cell viability, with plateaus at the bottom and top of the response, allowing the accurate determination of doxorubicin’s IC_50_ value (the concentration of doxorubicin required to reduce cell viability to 50%). HepG2 cells are the most widely used line for the toxicological screening of new drugs and are currently deployed in automated/high throughput systems.^56,57^ The ability to generate dose–response curves and calculate IC_50_ values using monolayer cryopreserved cells confirms their suitability for drug screening applications, finding biologically active compounds, and providing a way to increase automation through minimal cell-handling procedures.

HepG2 cytochrome p450 (CYP) activity was also assessed between nonfrozen and monolayer preserved cells 24 h post-thaw, Figure S16. CYP enzymes are vital in drug metabolism, with CYP3A4 being responsible for 50% of all drug metabolism.^81^ Thus, many drug screening applications rely on monitoring the inhibition of CYP enzymes, which could slow drug metabolism time resulting in undesired drug–drug interactions.^82,83^ Although insignificant, CYP3A4 activity of cells post-thaw was slightly suppressed post-thaw compared to nonfrozen cells, whereas CYP2C9 levels remained either unaffected or marginally higher. The innate expression of CYP enzymes was low for HepG2 cells and remained low even with the use of an inducer (rifampicin), Figure S17, which is consistent with previous studies.^84^ The selection of future cells with greater response to CYP inducers, with improved predictability for human hepatotoxin detection, will be explored to expand our library of cryopreserved monolayer cells. However, the data presented here clearly demonstrates that HepG2 monolayers can be cryopreserved in an assay-ready format, potentially enabling the first direct from storage to automated screening platform.

**Figure 10:**
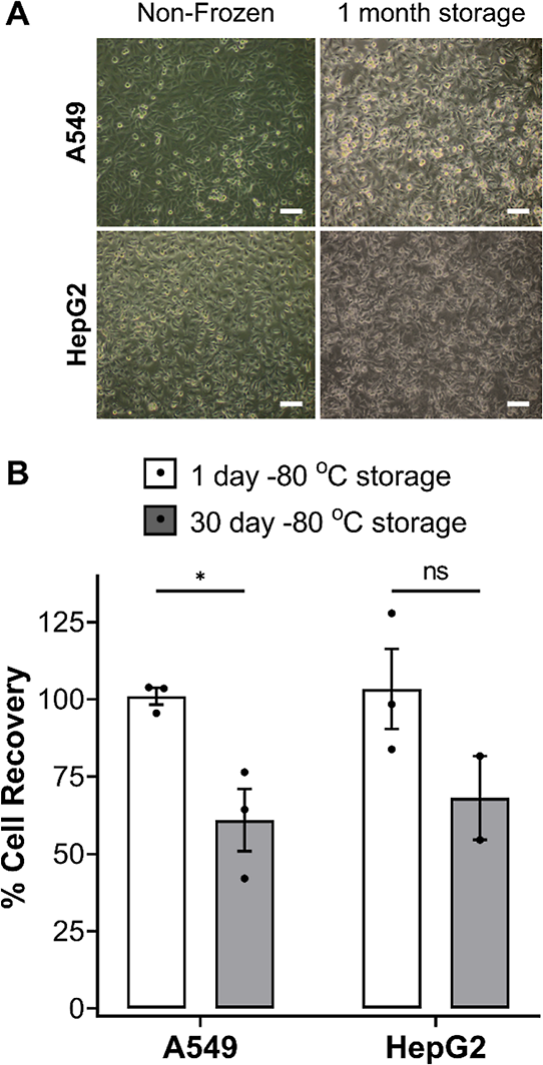
Long-term storage of cryopreserved cell monolayers. (A) Optical microscopy images were taken of A549 and HepG2 cell monolayers before freezing and 24 h post-thaw following 1 month of storage in a −80 °C freezer. Scale bar = 100 μm. (B) Average percentage cell recovery of A549 (white bar) and HepG2 (grey bar) cell monolayers following 1 and 30 days of storage in a −80 °C freezer were reported ± SEM from 2/3 independent repeats (ANOVA, Tukey PostHoc; ns: *p* > 0.05,*p ≤ 0.05).

To ensure that a sustainable supply of monolayer cryopreserved cell lines is possible, the long-term storage of cells is required. Although cells cryopreserved in suspension can be stored long-term at temperatures less than or equal to −80 °C, retaining >90% viability post-thaw,^8,85^ no reports were found on the medium-to-long term storage of cells cryopreserved as monolayers in a −80 °C freezer. Confluent A549 and HepG2 monolayers were cryopreserved as previously described and stored for 1 month in a −80 °C freezer. Optical microscopy images were taken of the confluent monolayers directly before freezing and 24 h post-thaw, Figure 10A. No changes to cell morphology were observed post-thaw and cells remained as confluent monolayers. A decrease in % cell recovery was found following long-term storage suggesting there may be some cell detachment during thawing or cell death, Figure 10B. However, no cell debris was found, and a cell recovery of ∼70% was obtained, which is sufficient to allow cells to be used for desired applications. Longer-term storage could be improved by conventional liquid N_2_ methods. Cells were also stored in dry ice for over 5 days, with no significant decrease in cell recovery (Figure S18), which provides a route for the transportation of cryopreserved plated cell monolayers.

## Conclusions

Here we introduce truly “assay-ready” cryopreserved adherent cells, ready to be used directly from a freezer within industry standard microplates, removing the need for standard handling, manipulations, management, and other time- and resource-expensive activities associated with cell culture and biomedical testing. This overcomes a historical challenge and perception that cells cannot be stored and distributed in monolayer format. The key component to enable such reproducible monolayer freezing was the use of a synthetic polyampholyte which, when used alongside the standard CPA DMSO, leads to dramatic increases in post-thaw cell yield and viability of up to 93%, compared to ∼50% using DMSO alone. Initial data suggests that the polyampholyte supports the cellular dehydration process during freezing, reducing the damaging effects of IIF. In addition to the macromolecular cryoprotectant, the freezing and thawing conditions were optimized to minimize well-to-well variance, ensuring homogenous cell recovery within and between microplates. Successful monolayer cryopreservation was demonstrated for A549, HepG2, and Caco-2 cells, representing a diverse range of commonly used cell lines within biomedical research and toxicology testing. To confirm that the cells were functional and healthy, a large panel of assays was undertaken. Post-thaw membrane damage and necrosis indicators were kept minimal and only a small increase in cells displaying apoptosis markers was observed. Cell cycle analysis and post-thaw growth curves confirmed that cells retained proliferative capacity. Cryopreserved A549 and HepG2 cell monolayers were subjected to a model assay development and toxicological challenge, to demonstrate that the cells are truly “assay ready” 24 h post-thaw and can be used for the testing of pharmaceutically active compounds. Overall, we have demonstrated that cells can be cryopreserved as “assay-ready” monolayers, in a scalable and versatile manner, which will enable the development of new screening technologies, enhance automation, and reduce the experimental burden on researchers.

## Data Access Statement

The research data supporting this publication is found in the Supporting Information and any additional data can be found at http://wrap.warwick.ac.uk.

## Supporting Information Available

Polymer characterization data; additional cell recovery data, live/dead images and caspase images; resazurin cell viability assay development; growth curve measurements; cell cycle flow cytometry histograms; HepG2 cytochrome p450 activity assays; cell recovery after dry ice storage; and cryomicroscopy images (PDF)

## References

[ref1] Mazur P.; Leibo S. P.; Chu E. H. Y. A two-factor hypothesis of freezing injury. Exp. Cell Res. 1972, 71, 345–355. 10.1016/0014-4827(72)90303-5. 5045639

[ref2] Lovelock J. E.; Bishop M. W. Prevention of Freezing Damage to Living Cells by Dimethyl Sulphoxide. Nature 1959, 183, 1394–1395. 10.1038/1831394a0. 13657132

[ref3] Cheng C. Y.; Song J.; Pas J.; Meijer L. H. H.; Han S. DMSO Induces Dehydration near Lipid Membrane Surfaces. Biophys. J. 2015, 109, 330–339. 10.1016/j.bpj.2015.06.011. 26200868 PMC4621616

[ref4] Gurtovenko A. A.; Anwar J. Modulating the Structure and Properties of Cell Membranes: The Molecular Mechanism of Action of Dimethyl Sulfoxide. J. Phys. Chem. B 2007, 111, 10453–10460. 10.1021/jp073113e. 17661513

[ref5] Hoon T.; Choel S.; Hyun J.; Yoon J.; Hong J.; Seo U.; Won C.; Ryul S.; Han J. Cryopreservation and Its Clinical Applications. Integr. Med. Res. 2017, 6, 12–18. 10.1016/j.imr.2016.12.001. 28462139 PMC5395684

[ref6] Murray K. A.; Gibson M. I. Post-Thaw Culture and Measurement of Total Cell Recovery Is Crucial in the Evaluation of New Macromolecular Cryoprotectants. Biomacromolecules 2020, 21, 2864–2873. 10.1021/acs.biomac.0c00591. 32501710 PMC7362331

[ref7] Tomás R. M. F.; Bailey T. L.; Hasan M.; Gibson M. I. Extracellular Antifreeze Protein Significantly Enhances the Cryopreservation of Cell Monolayers. Biomacromolecules 2019, 20, 3864–3872. 10.1021/acs.biomac.9b00951. 31498594 PMC6794639

[ref8] Miyamoto Y.; Ikeuchi M.; Noguchi H.; Hayashi S. Long-term Cryopreservation of Human and other Mammalian Cells at -80 °C for 8 Years. Cell Med. 2018, 10, 2155179017733148 10.1177/2155179017733148. 32634179 PMC6172990

[ref9] Urbina M. A.; Watts A. J. R.; Reardon E. E. Labs Should Cut Plastic Waste Too. Nature 2015, 528, 479 10.1038/528479c. 26701046

[ref10] Geraghty R. J.; Capes-Davis A.; Davis J. M.; Downward J.; Freshney R. I.; Knezevic I.; Lovell-Badge R.; Masters J. R.; Meredith J.; Stacey G. N.; et al. Guidelines for the Use of Cell Lines in Biomedical Research. Br. J. Cancer 2014, 111, 1021–1046. 10.1038/bjc.2014.166. 25117809 PMC4453835

[ref11] Joddar B.; Hoshiba T.; Chen G.; Ito Y. Stem Cell Culture Using Cell-Derived Substrates. Biomater. Sci. 2014, 2, 1595–1603. 10.1039/c4bm00126e. 32481944

[ref12] Trajkovic K.; Valdez C.; Ysselstein D.; Krainc D. Fluctuations in Cell Density Alter Protein Markers of Multiple Cellular Compartments, Confounding Experimental Outcomes. PLoS One 2019, 14, e0211727 10.1371/journal.pone.0211727. 30716115 PMC6361456

[ref13] Yamasaki C.; Ishida Y.; Yanagi A.; Yoshizane Y.; Kojima Y.; Ogawa Y.; Kageyama Y.; Iwasaki Y.; Ishida S.; Chayama K.; et al. Culture Density Contributes to Hepatic Functions of Fresh Human Hepatocytes Isolated from Chimeric Mice with Humanized Livers: Novel, Long-Term, Functional Two-Dimensional in Vitro Tool for Developing New Drugs. PLoS One 2020, 15, e0237809 10.1371/journal.pone.0237809. 32915792 PMC7485858

[ref14] Gómez S.; Del Mont Llosas M.; Verdú J.; Roura S.; Lloreta J.; Fabre M.; García De Herreros A. Independent Regulation of Adherens and Tight Junctions by Tyrosine Phosphorylation in Caco-2 Cells. Biochim. Biophys. Acta, Mol. Cell Res. 1999, 1452, 121–132. 10.1016/s0167-4889(99)00124-x. 10559465

[ref15] Ma W.; O’Shaughnessy T.; Chang E. Cryopreservation of Adherent Neuronal Networks. Neurosci. Lett. 2006, 403, 84 10.1016/j.neulet.2006.04.064. 16759804

[ref16] Pless-Petig G.; Knoop S.; Rauen U. Serum- and Albumin-Free Cryopreservation of Endothelial Monolayers with a New Solution. Organogenesis 2018, 14, 107–121. 10.1080/15476278.2018.1501136. 30081735 PMC6150062

[ref17] Acker J. P.; McGann L. E. Cell-Cell Contact Affects Membrane Integrity after Intracellular Freezing. Cryobiology 2000, 40, 54–63. 10.1006/cryo.1999.2221. 10679150

[ref18] Higgins A. Z.; Karlsson J. O. M. Effects of Intercellular Junction Protein Expression on Intracellular Ice Formation in Mouse Insulinoma Cells. Biophys. J. 2013, 105, 2006–2015. 10.1016/j.bpj.2013.09.028. 24209845 PMC3824721

[ref19] Acker J. P.; Elliott J. A. W.; McGann L. E. Intercellular Ice Propagation: Experimental Evidence for Ice Growth through Membrane Pores. Biophys. J. 2001, 81, 1389–1397. 10.1016/s0006-3495(01)75794-3. 11509353 PMC1301618

[ref20] Acker J. P.; Larese A.; Yang H.; Petrenko A.; McGann L. E. Intracellular Ice Formation Is Affected by Cell Interactions. Cryobiology 1999, 38, 363–371. 10.1006/cryo.1999.2179. 10413578

[ref21] Bahari L.; Bein A.; Yashunsky V.; Braslavsky I. Directional Freezing for the Cryopreservation of Adherent Mammalian Cells on a Substrate. PLoS One 2018, 13, e0192265 10.1371/journal.pone.0192265. 29447224 PMC5813933

[ref22] Daily M. I.; Whale T. F.; Partanen R.; Harrison A. D.; Kilbride P.; Lamb S.; Morris G. J.; Picton H. M.; Murray B. J. Cryopreservation of Primary Cultures of Mammalian Somatic Cells in 96-Well Plates Benefits from Control of Ice Nucleation. Cryobiology 2020, 93, 62–69. 10.1016/j.cryobiol.2020.02.008. 32092295 PMC7191264

[ref23] Murray K. A.; Kinney N. L. H.; Griffiths C. A.; Hasan M.; Gibson M. I.; Whale T. F. Pollen Derived Macromolecules Serve as a New Class of Ice-Nucleating Cryoprotectants. Sci. Rep. 2022, 12, 12295 10.1038/s41598-022-15545-4. 35854036 PMC9296471

[ref24] Balcerzak A. K.; Capicciotti C. J.; Briard J. G.; Ben R. N. Designing Ice Recrystallization Inhibitors: From Antifreeze (Glyco)Proteins to Small Molecules. RSC Adv. 2014, 4, 42682–42696. 10.1039/c4ra06893a.

[ref25] Wu L. K.; Tokarew J. M.; Chaytor J. L.; von Moos E.; Li Y.; Palii C.; Ben R. N.; Allan D. S. Carbohydrate-Mediated Inhibition of Ice Recrystallization in Cryopreserved Human Umbilical Cord Blood. Carbohydr. Res. 2011, 346, 86–93. 10.1016/j.carres.2010.10.016. 21075361

[ref26] Capicciotti C. J.; Poisson J. S.; Boddy C. N.; Ben R. N. Modulation of Antifreeze Activity and the Effect upon Post-Thaw HepG2 Cell Viability after Cryopreservation. Cryobiology 2015, 70, 79–89. 10.1016/j.cryobiol.2015.01.002. 25595636

[ref27] Graham B.; Bailey T. L.; Healey J. R. J.; Marcellini M.; Deville S.; Gibson M. I. Polyproline as a Minimal Antifreeze Protein Mimic That Enhances the Cryopreservation of Cell Monolayers. Angew. Chem., Int. Ed. 2017, 129, 16157–16160. 10.1002/ange.201706703. PMC5722203 29044869

[ref28] Briard J. G.; Poisson J. S.; Turner T. R.; Capicciotti C. J.; Acker J. P.; Ben R. N. Small Molecule Ice Recrystallization Inhibitors Mitigate Red Blood Cell Lysis during Freezing, Transient Warming and Thawing. Sci. Rep. 2016, 6, 23619 10.1038/srep23619. 27021850 PMC4810524

[ref29] Deller R. C.; Pessin J. E.; Vatish M.; Mitchell D. A.; Gibson M. I. Enhanced Non-Vitreous Cryopreservation of Immortalized and Primary Cells by Ice-Growth Inhibiting Polymers. Biomater. Sci. 2016, 4, 1079–1084. 10.1039/c6bm00129g. 27152370 PMC4918798

[ref30] Deller R. C.; Vatish M.; Mitchell D. A.; Gibson M. I. Synthetic Polymers Enable Non-Vitreous Cellular Cryopreservation by Reducing Ice Crystal Growth during Thawing. Nat. Commun. 2014, 5, 3244 10.1038/ncomms4244. 24488146

[ref31] Carpenter J. F.; Hansen T. N. Antifreeze Protein Modulates Cell Survival during Cryopreservation: Mediation through Influence on Ice Crystal Growth. Proc. Natl. Acad. Sci. U.S.A. 1992, 89, 8953–8957. 10.1073/pnas.89.19.8953. 1409591 PMC50042

[ref32] Biggs C. I.; Bailey T. L.; Ben Graham B.; Stubbs C.; Fayter A.; Gibson M. I. Polymer Mimics of Biomacromolecular Antifreezes. Nat. Commun. 2017, 8, 1546 10.1038/s41467-017-01421-7. 29142216 PMC5688100

[ref33] Murray K. A.; Gibson M. I. Chemical Approaches to Cryopreservation. Nat. Rev. Chem. 2022, 6, 579–593. 10.1038/s41570-022-00407-4. 35875681 PMC9294745

[ref34] Chao H.; Davies P. L.; Carpenter J. F. Effects of Antifreeze Proteins on Red Blood Cell Survival during Cryopreservation. J. Exp. Biol. 1996, 199, 2071–2076. 10.1242/jeb.199.9.2071. 8831147

[ref35] Geng H.; Liu X.; Shi G.; Bai G.; Ma J.; Chen J.; Wu Z.; Song Y.; Fang H.; Wang J. Graphene Oxide Restricts Growth and Recrystallization of Ice Crystals. Angew. Chem., Int. Ed. 2017, 56, 997–1001. 10.1002/anie.201609230. 27976493

[ref36] Bailey T. L.; Hernandez-Fernaud J. R.; Gibson M. I. Proline Pre-Conditioning of Cell Monolayers Increases Post-Thaw Recovery and Viability by Distinct Mechanisms to Other Osmolytes. RSC Med. Chem. 2021, 12, 982–993. 10.1039/d1md00078k. 34223163 PMC8221256

[ref37] Tomás R. M. F.; Bailey T. L.; Hasan M.; Gibson M. I. Extracellular Antifreeze Protein Significantly Enhances the Cryopreservation of Cell Monolayers. Biomacromolecules 2019, 20, 3864–3872. 10.1021/acs.biomac.9b00951. 31498594 PMC6794639

[ref38] Stubbs C.; Bailey T. L.; Murray K.; Gibson M. I. Polyampholytes as Emerging Macromolecular Cryoprotectants. Biomacromolecules 2020, 21, 7–17. 10.1021/acs.biomac.9b01053. 31418266 PMC6960013

[ref39] Bailey T. L.; Wang M.; Solocinski J.; Nathan B. P.; Chakraborty N.; Menze M. A. Protective Effects of Osmolytes in Cryopreserving Adherent Neuroblastoma (Neuro-2a) Cells. Cryobiology 2015, 71, 472–480. 10.1016/j.cryobiol.2015.08.015. 26408850

[ref40] Stokich B.; Osgood Q.; Grimm D.; Moorthy S.; Chakraborty N.; Menze M. A. Cryopreservation of Hepatocyte (HepG2) Cell Monolayers: Impact of Trehalose. Cryobiology 2014, 69, 281–290. 10.1016/j.cryobiol.2014.08.001. 25127872

[ref41] Matsumura K.; Hyon S. H. Polyampholytes as Low Toxic Efficient Cryoprotective Agents with Antifreeze Protein Properties. Biomaterials 2009, 30, 4842–4849. 10.1016/j.biomaterials.2009.05.025. 19515417

[ref42] Vorontsov D. A.; Sazaki G.; Hyon S. H.; Matsumura K.; Furukawa Y. Antifreeze Effect of Carboxylated ε-Poly-l-lysine on the Growth Kinetics of Ice Crystals. J. Phys. Chem. B 2014, 118, 10240–10249. 10.1021/jp507697q. 25113284

[ref43] Matsumura K.; Hayashi F.; Nagashima T.; Rajan R.; Hyon S.-H. Molecular Mechanisms of Cell Cryopreservation with Polyampholytes Studied by Solid-State NMR. Commun. Mater. 2021, 2, 15 10.1038/s43246-021-00118-1.

[ref44] Rajan R.; Hayashi F.; Nagashima T.; Matsumura K. Toward a Molecular Understanding of the Mechanism of Cryopreservation by Polyampholytes: Cell Membrane Interactions and Hydrophobicity. Biomacromolecules 2016, 17, 1882–1893. 10.1021/acs.biomac.6b00343. 27077533

[ref45] Tomczak M. M.; Hincha D. K.; Estrada S. D.; Feeney R. E.; Crowe J. H. Antifreeze Proteins Differentially Affect Model Membranes during Freezing. Biochim. Biophys. Acta, Biomembr. 2001, 1511, 255–263. 10.1016/s0005-2736(01)00281-4. 11286968

[ref46] Watanabe H.; Kohaya N.; Kamoshita M.; Fujiwara K.; Matsumura K.; Hyon S. H.; Ito J.; Kashiwazaki N. Efficient Production of Live Offspring from Mouse Oocytes Vitrified with a Novel Cryoprotective Agent, Carboxylated ε-Poly-L-Lysine. PLoS One 2013, 8, e83613 10.1371/journal.pone.0083613. 24376724 PMC3871522

[ref47] Maehara M.; Sato M.; Watanabe M.; Matsunari H.; Kokubo M.; Kanai T.; Sato M.; Matsumura K.; Hyon S. H.; Yokoyama M.; et al. Development of a Novel Vitrification Method for Chondrocyte Sheets. BMC Biotechnol. 2013, 13, 58 10.1186/1472-6750-13-58. 23886356 PMC3726287

[ref48] Matsumura K.; Kawamoto K.; Takeuchi M.; Yoshimura S.; Tanaka D.; Hyon S.-H. H. Cryopreservation of a Two-Dimensional Monolayer Using a Slow Vitrification Method with Polyampholyte to Inhibit Ice Crystal Formation. ACS Biomater. Sci. Eng. 2016, 2, 1023–1029. 10.1021/acsbiomaterials.6b00150. 33429511

[ref49] Matsumura K.; Hayashi F.; Nagashima T.; Hyon S. H. Long-Term Cryopreservation of Human Mesenchymal Stem Cells Using Carboxylated Poly-l-Lysine without the Addition of Proteins or Dimethyl Sulfoxide. J. Biomater. Sci., Polym. Ed. 2013, 24, 1484–1497. 10.1080/09205063.2013.771318. 23829460

[ref50] Matsumura K.; Bae J. Y.; Hyon S. H. Polyampholytes as Cryoprotective Agents for Mammalian Cell Cryopreservation. Cell Transplant. 2010, 19, 691–699. 10.3727/096368910x508780. 20525437

[ref51] Stubbs C.; Murray K. A.; Ishibe T.; Mathers R. T.; Gibson M. I. Combinatorial Biomaterials Discovery Strategy to Identify New Macromolecular Cryoprotectants. ACS Macro Lett. 2020, 9, 290–294. 10.1021/acsmacrolett.0c00044. 32337092 PMC7175595

[ref52] Zhao J.; Johnson M. A.; Fisher R.; Burke N. A. D.; Stöver H. D. H. Synthetic Polyampholytes as Macromolecular Cryoprotective Agents. Langmuir 2019, 35, 1807–1817. 10.1021/acs.langmuir.8b01602. 30134094

[ref53] Rajan R.; Jain M.; Matsumura K. Cryoprotective Properties of Completely Synthetic Polyampholytes via Reversible Addition-Fragmentation Chain Transfer (RAFT) Polymerization and the Effects of Hydrophobicity. J. Biomater. Sci., Polym. Ed. 2013, 24, 1767 10.1080/09205063.2013.801703. 23721063

[ref54] Bailey T. L.; Stubbs C.; Murray K.; Tomás R. M. F.; Otten L.; Gibson M. I. Synthetically Scalable Poly(ampholyte) Which Dramatically Enhances Cellular Cryopreservation. Biomacromolecules 2019, 20, 3104–3114. 10.1021/acs.biomac.9b00681. 31268698 PMC6692820

[ref55] Murray K. A.; Tomás R. M. F.; Gibson M. I. Low DMSO Cryopreservation of Stem Cells Enabled by Macromolecular Cryoprotectants. ACS Appl. Bio Mater. 2020, 3, 5627–5632. 10.1021/acsabm.0c00638. PMC7509910 32984779

[ref56] Soldatow V. Y.; LeCluyse E. L.; Griffith L. G.; Rusyn I. In vitro models for liver toxicity testing. Toxicol. Res. 2013, 2, 23–39. 10.1039/c2tx20051a. PMC3593300 23495363

[ref57] Bale S. S.; Vernetti L.; Senutovitch N.; Jindal R.; Hegde M.; Gough A.; McCarty W. J.; Bakan A.; Bhushan A.; Shun T. Y.; et al. In Vitro Platforms for Evaluating Liver Toxicity. Exp. Biol. Med. 2014, 239, 1180–1191. 10.1177/1535370214531872. PMC4156546 24764241

[ref58] Acker J. P.; Croteau I. M. Pre- and Post-Thaw Assessment of Intracellular Ice Formation. J. Microsc. 2004, 215, 131–138. 10.1111/j.0022-2720.2004.01375.x. 15315499

[ref59] Pozarowski P.; Darzynkiewicz Z. Analysis of Cell Cycle by Flow Cytometry. Methods Mol. Biol. 2004, 281, 301 10.1385/1-59259-811-0:301. 15220539

[ref60] Acker J. P.; Mcgann L. E. Innocuous Intracellular Ice Improves Survival of Frozen Cells. Cell Transplant. 2002, 11, 563–571. 10.3727/000000002783985468. 12428746

[ref61] Meryman H. T. Physical Limitations of the Rapid Freezing Method. Proc. R. Soc. London 1957, 147, 452–459. 10.1098/rspb.1957.0064. 13494458

[ref62] Palchaudhuri R.; Lambrecht M. J.; Botham R. C.; Partlow K. C.; van Ham T. J.; Putt K. S.; Nguyen L. T.; Kim S. H.; Peterson R. T.; Fan T. M.; et al. A Small Molecule That Induces Intrinsic Pathway Apoptosis with Unparalleled Speed. Cell Rep. 2015, 13, 2027–2036. 10.1016/j.celrep.2015.10.042. 26655912 PMC4683402

[ref63] Goldstein J. C.; Kluck R. M.; Green D. R. A single cell analysis of apoptosis. Ordering the apoptotic phenotype. Ann. N.Y. Acad. Sci. 2000, 926, 132 10.1111/j.1749-6632.2000.tb05607.x. 11193030

[ref64] Hernández-Tapia L. G.; Fohlerová Z.; Žídek J.; Alvarez-Perez M. A.; Ĉelko L.; Kaiser J.; Montufar E. B. Effects of Cryopreservation on Cell Metabolic Activity and Function of Biofabricated Structures Laden with Osteoblasts. Materials 2020, 13, 1966 10.3390/ma13081966. PMC7215951 32331435

[ref65] Rodrigues A. Q.; Picolo V. L.; Goulart J. T.; Silva I. M. G.; Ribeiro R. B.; Aguiar B. A.; Ferreira Y. B.; Oliveira D. M.; Lucci C. M.; de Bem A. F.; et al. Metabolic Activity in Cryopreserved and Grafted Ovarian Tissue Using High-Resolution Respirometry. Sci. Rep. 2021, 11, 21517 10.1038/s41598-021-01082-z. 34728762 PMC8563867

[ref66] Bahsoun S.; Coopman K.; Akam E. C. Quantitative Assessment of the Impact of Cryopreservation on Human Bone Marrow-Derived Mesenchymal Stem Cells: Up to 24 h Post-Thaw and Beyond. Stem Cell Res. Ther. 2020, 11, 540 10.1186/s13287-020-02054-2. 33317625 PMC7734731

[ref67] Reynolds P. M.; Rasmussen C. H.; Hansson M.; Dufva M.; Riehle M. O.; Gadegaard N. Controlling Fluid Flow to Improve Cell Seeding Uniformity. PLoS One 2018, 13, e0207211 10.1371/journal.pone.0207211. 30440053 PMC6237340

[ref68] Hewitt N. J.; Hewitt P. Phase I and II Enzyme Characterization of Two Sources of HepG2 Cell Lines. Xenobiotica 2004, 34, 243–256. 10.1080/00498250310001657568. 15204697

[ref69] Tolosa L.; Gómez-Lechón M. J.; Pérez-Cataldo G.; Castell J. V.; Donato M. T. HepG2 Cells Simultaneously Expressing Five P450 Enzymes for the Screening of Hepatotoxicity: Identification of Bioactivable Drugs and the Potential Mechanism of Toxicity Involved. Arch. Toxicol. 2013, 87, 1115–1127. 10.1007/s00204-013-1012-x. 23397584

[ref70] Bissoyi A.; Pramanik K. Role of the Apoptosis Pathway in Cryopreservation-Induced Cell Death in Mesenchymal Stem Cells Derived from Umbilical Cord Blood. Biopreserv. Biobanking 2014, 12, 246–254. 10.1089/bio.2014.0005. 25162461

[ref71] Riedl S. J.; Shi Y. Molecular Mechanisms of Caspase Regulation during Apoptosis. Nat. Rev. Mol. Cell Biol. 2004, 5, 897–907. 10.1038/nrm1496. 15520809

[ref72] Schmidt-Mende J.; Hellström-Lindberg E.; Joseph B.; Zhivotovsky B. Freezing Induces Artificial Cleavage of Apoptosis-Related Proteins in Human Bone Marrow Cells. J. Immunol. Methods 2000, 245, 91 10.1016/s0022-1759(00)00285-4. 11042286

[ref73] Mathew A. J.; Van Buskirk R. G.; Baust J. G. Improved Hypothermic Preservation of Human Renal Cells through Suppression of Both Apoptosis and Necrosis. Cell Preserv. Technol. 2003, 1, 239–253. 10.1089/15383440260682071.

[ref74] Li X.; Krawetz R.; Liu S.; Meng G.; Rancourt D. E. ROCK Inhibitor Improves Survival of Cryopreserved Serum/Feeder-Free Single Human Embryonic Stem Cells. Hum. Reprod. 2009, 24, 580 10.1093/humrep/den404. 19056770

[ref75] Norouzzadeh M.; Kalikias Y.; Mohamadpur Z.; Sharifi L. Determining Population Doubling Time and the Appropriate Number of HepG2 Cells for Culturing in 6- Well Plate. Int. Res. J. Appl. Basic Sci. 2016, 10, 299–303.

[ref76] Devine R. D.; Sekhri P.; Behbehani G. K. Effect of Storage Time and Temperature on Cell Cycle Analysis by Mass Cytometry. Cytometry, Part A 2018, 93, 1141–1149. 10.1002/cyto.a.23630. 30378741

[ref77] Darzynkiewicz Z.; Bedner E.; Smolewski P. Flow Cytometry in Analysis of Cell Cycle and Apoptosis. Semin. Hematol. 2001, 38, 179–193. 10.1053/shem.2001.21929. 11309699

[ref78] Liu J. D.; Wang Y. J.; Chen C. H.; Yu C. F.; Chen L. C.; Lin J. K.; Liang Y. C.; Lin S. Y.; Ho Y. S. Molecular Mechanisms of G0/G1 Cell-Cycle Arrest and Apoptosis Induced by Terfenadine in Human Cancer Cells. Mol. Carcinog. 2003, 37, 39–50. 10.1002/mc.10118. 12720299

[ref79] Khamchun S.; Thongboonkerd V. Cell Cycle Shift from G0/G1 to S and G2/M Phases Is Responsible for Increased Adhesion of Calcium Oxalate Crystals on Repairing Renal Tubular Cells at Injured Site. Cell Death Discovery 2018, 4, 106 10.1038/s41420-018-0123-9. 30774989 PMC6374384

[ref80] Liu Q.; Cao Y.; Zhou P.; Gui S.; Wu X.; Xia Y.; Tu J. Panduratin A Inhibits Cell Proliferation by Inducing G0/G1 Phase Cell Cycle Arrest and Induces Apoptosis in Breast Cancer Cells. Biomol. Ther. 2018, 26, 328–334. 10.4062/biomolther.2017.042. PMC5933901 29301388

[ref81] Teo Y. L.; Ho H. K.; Chan A. Metabolism-related pharmacokinetic drug–drug interactions with tyrosine kinase inhibitors: current understanding, challenges and recommendations. Br. J. Clin. Pharmacol. 2015, 79, 241–253. 10.1111/bcp.12496. 25125025 PMC4309630

[ref82] Sevrioukova I. Interaction of Human Drug-Metabolizing CYP3A4 with Small Inhibitory Molecules. Biochemistry 2019, 58, 930–939. 10.1021/acs.biochem.8b01221. 30676743 PMC6448587

[ref83] Hukkanen J. Induction of cytochrome P450 enzymes: a view on humanin vivofindings. Expet Rev. Clin. Pharmacol. 2012, 5, 569–585. 10.1586/ecp.12.39. 23121279

[ref84] Gerets H. H. J.; Tilmant K.; Gerin B.; Chanteux H.; Depelchin B. O.; Dhalluin S.; Atienzar F. A. Characterization of Primary Human Hepatocytes, HepG2 Cells, and HepaRG Cells at the MRNA Level and CYP Activity in Response to Inducers and Their Predictivity for the Detection of Human Hepatotoxins. Cell Biol. Toxicol. 2012, 28, 69–87. 10.1007/s10565-011-9208-4. 22258563 PMC3303072

[ref85] Winter J. M.; Jacobson P.; Bullough B.; Christensen A. P.; Boyer M.; Reems J. A. Long-Term Effects of Cryopreservation on Clinically Prepared Hematopoietic Progenitor Cell Products. Cytotherapy 2014, 16, 965–975. 10.1016/j.jcyt.2014.02.005. 24910385

