# Supplementary material for: Assay-ready Cryopreserved Cell Monolayers Enabled by Macromolecular Cryoprotectants
Source: Biomacromolecules. 2022 Aug 16;23(9):3948–59. doi: 10.1021/acs.biomac.2c00791 (PMC9472225; doi:10.1021/acs.biomac.2c00791)
Supplement: Supplementary file 1 — bm2c00791_si_001.pdf [file bm2c00791_si_001.pdf]

Electronic Supporting Information

Assay-Ready Cryopreserved Cell Monolayers Enabled by Macromolecular Cryoprotectants

Ruben M. F. Tomás,[a] Akalabya Bissoyi,[a] Thomas R. Congdon,[c] and Matthew I. Gibson[a,b*]

[a] *Division of Biomedical Sciences, Warwick Medical School, University of Warwick, Gibbet Hill Road, Coventry, CV4 7AL, UK;*

[b] *Department of Chemistry, University of Warwick, Gibbet Hill Road, Coventry, CV4 7AL, UK;*

[c]*Cryologyx Ltd, 71-75 Shelton Street, London, UK, WC2H 9JQ, UK*

*Corresponding Author m.i.gibson@warwick.ac.uk

1 Additional Data

1.1 Polymer Characterisation

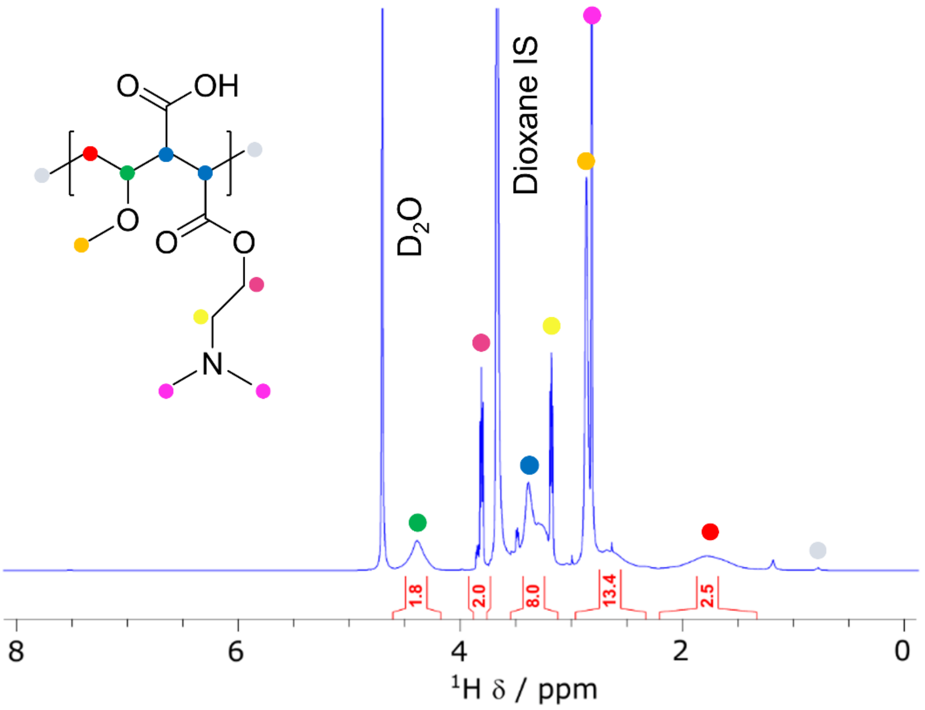

Figure S1. ^{1}H NMR characterisation of polyampholyte. IS = internal standard.

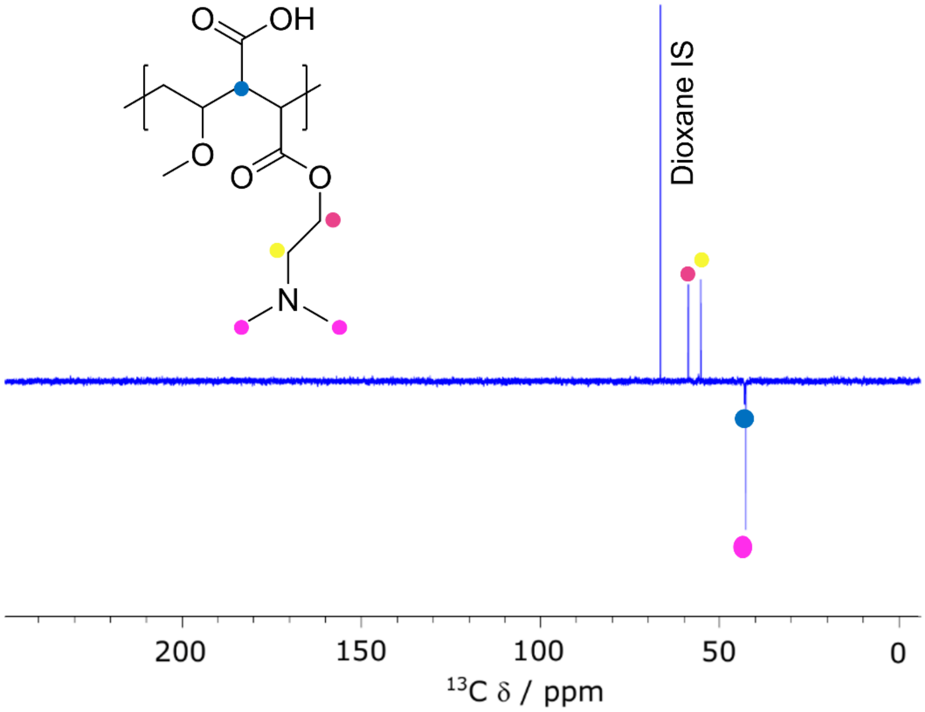

Figure S2. ^{13}C NMR characterisation of polyampholyte. IS = internal standard.

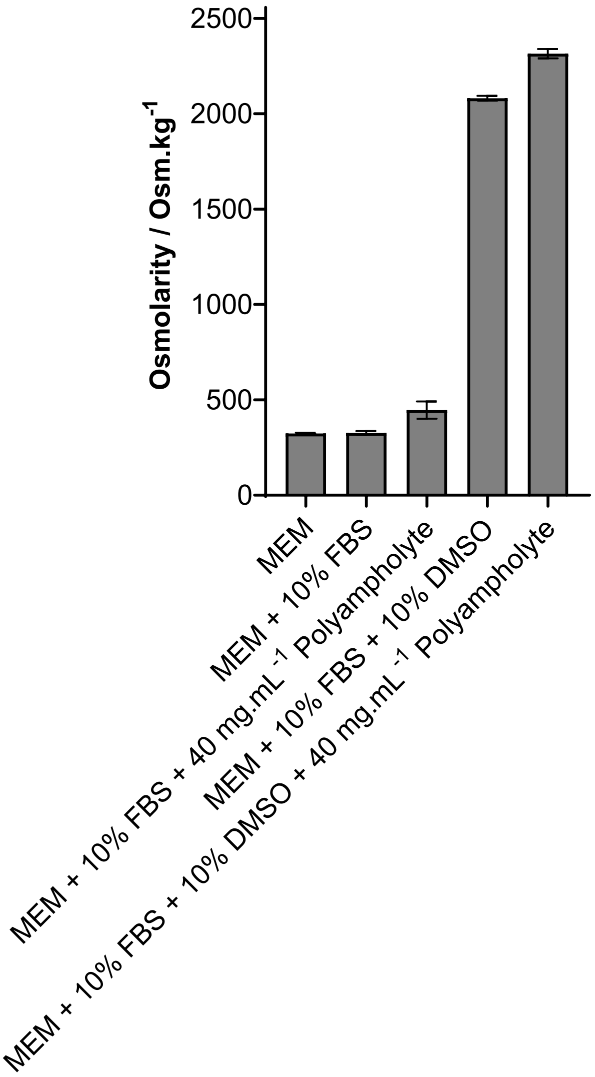

Figure S3. Osmolarity measurements of Minimum Essential Medium Eagle supplemented with 10%v/v FBS, 10 %v/v DMSO and/or polyampholyte (40 mg.mL^{-1}).

1.2 Cell Recovery Data

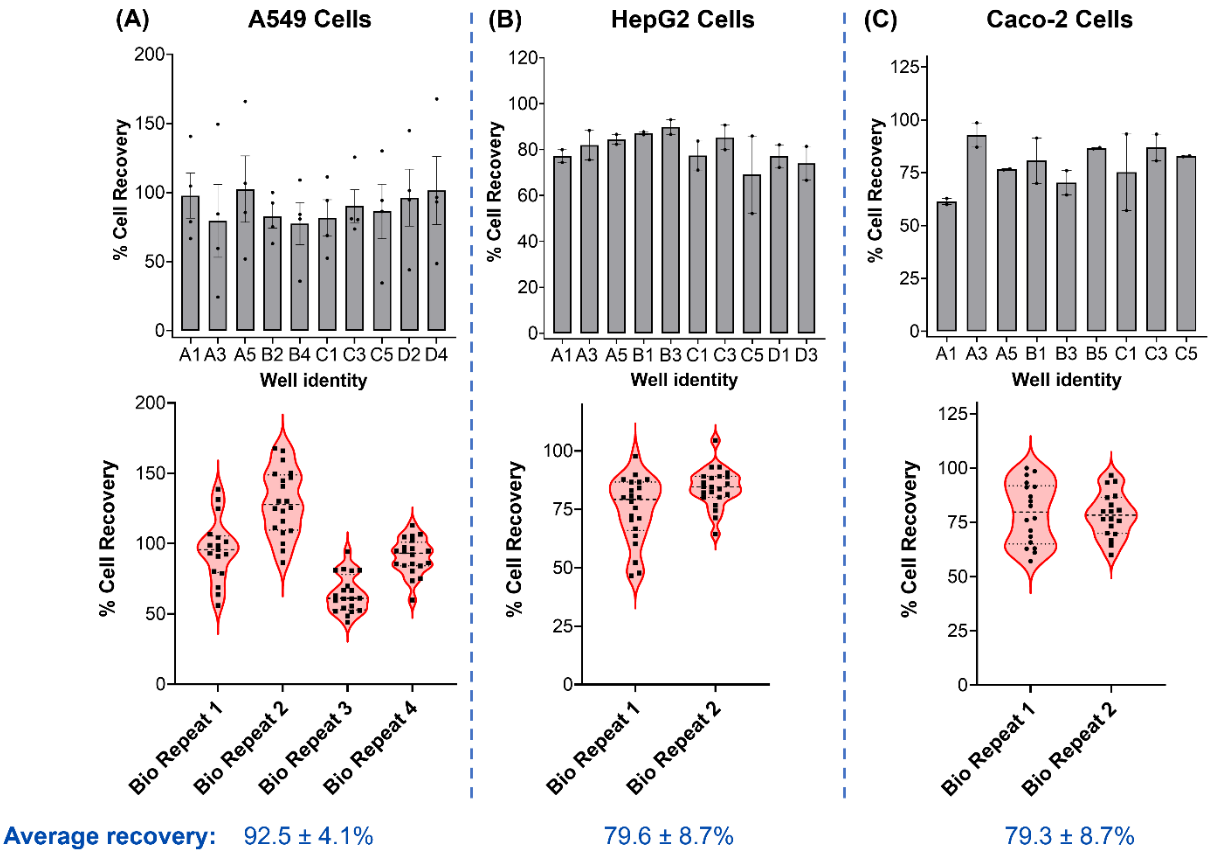

Figure S4. Post-thaw cell recovery of (**A**) A549, (**B**) HepG2 and (**C**) Caco-2 confluent monolayers cryopreserved with 40 mg.mL^{-1} of polyampholyte and 10 %v/v DMSO. Cell counts were completed 24 h post-thaw. Graphs have been plotted as bar graphs and violin plots to show average cell recoveries and well-to-well variability. Data is presented as mean % cell recovery ± SEM from two independent repeats.

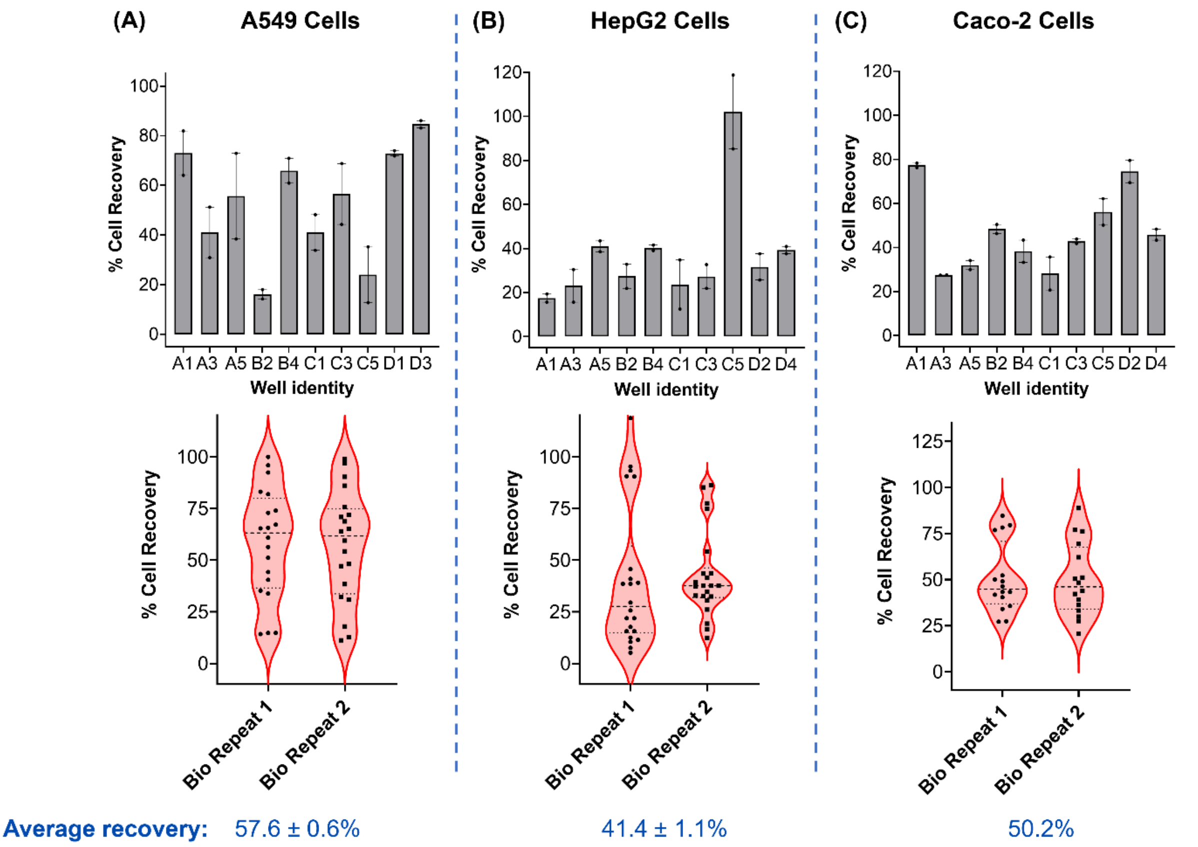

Figure S5. Post-thaw cell recovery of (**A**) A549, (**B**) HepG2 and (**C**) Caco-2 confluent monolayers cryopreserved with 10 %v/v DMSO. Cell counts were completed 24 h post-thaw. Graphs have been plotted as bar graphs and violin plots to show average cell recoveries and well-to-well variability. Data is presented as mean % cell recovery ± SEM from two independent repeats.

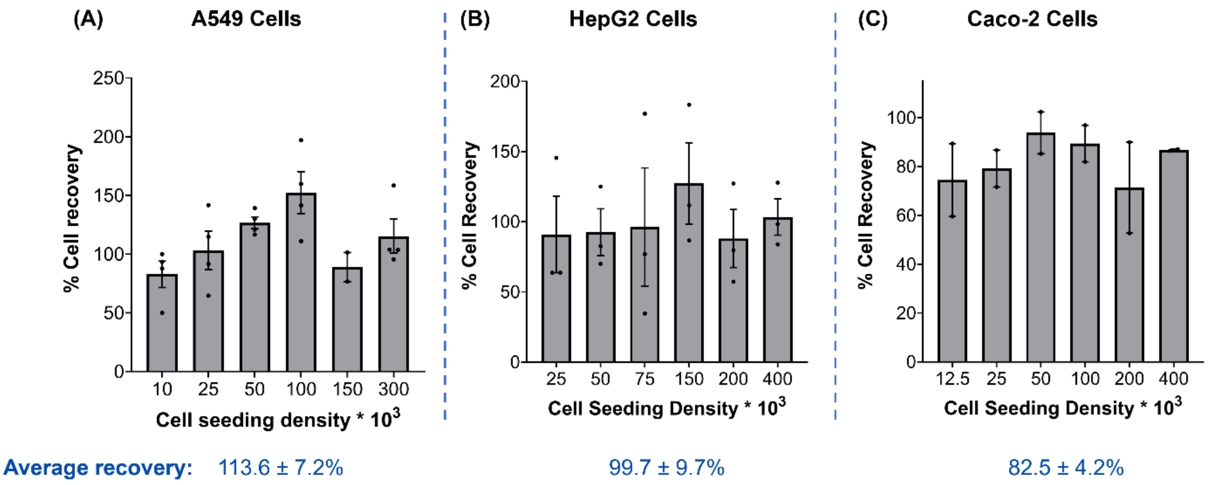

Figure S6. Percentage cell recovery of (**A**) A549, (**B**) HepG2 and (**C**) Caco-2 cells plated at different cell densities frozen with 40 mg.mL^{-1} of polyampholyte and 10% DMSO. Cell counts were completed 24 h post-thaw. Data is presented as mean % cell recovery ± SEM from two to four independent repeats.

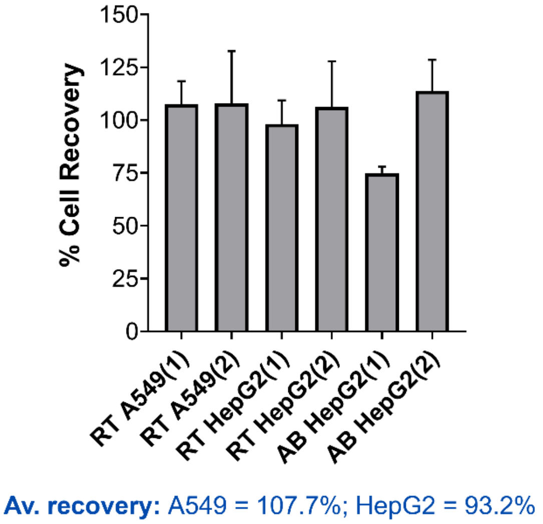

Figure S7. Scalable freezing process. Six plates containing A549 and HepG2 cells were frozen with 40 mg.mL^{-1} of polyampholyte and 10 %v/v DMSO by two independent scientists (Ruben Tomas, RT; Akalabya Bissoyi, AB), simultaneously, and the mean % cell recovery was determined 24 h post-thaw ± SEM of five technical repeats (different wells from the same well plate).

1.3 Before and after freezing imaging

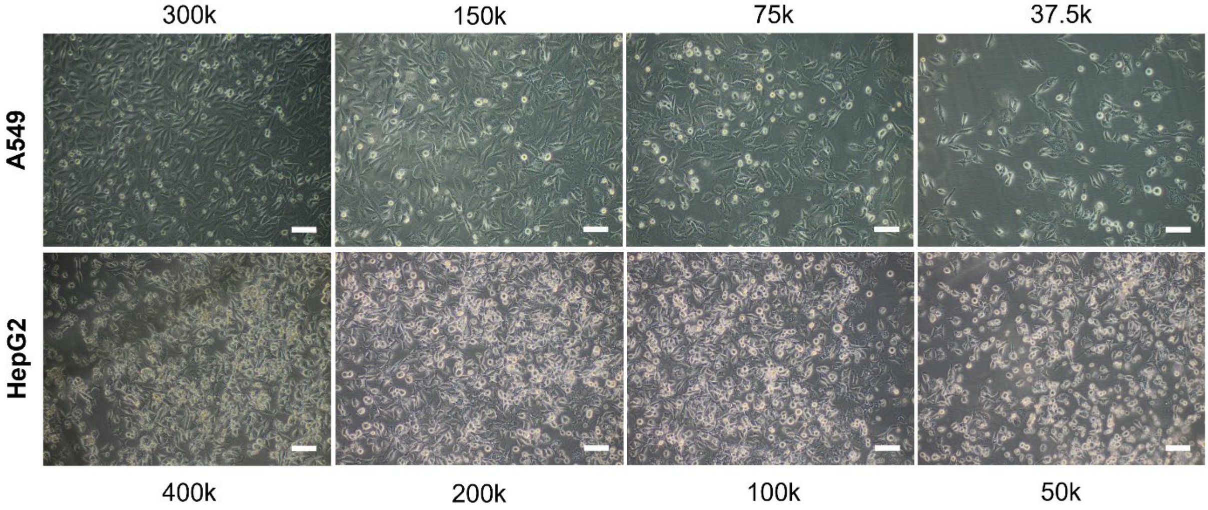

Figure S8. Post-thaw images of cells frozen at different cell densities. A459 and HepG2 cells were frozen with 10% DMSO and 40 mg.mL^{-1} of polyampholyte and images were taken 24 h post-thaw. Scale bar = 100 µm.

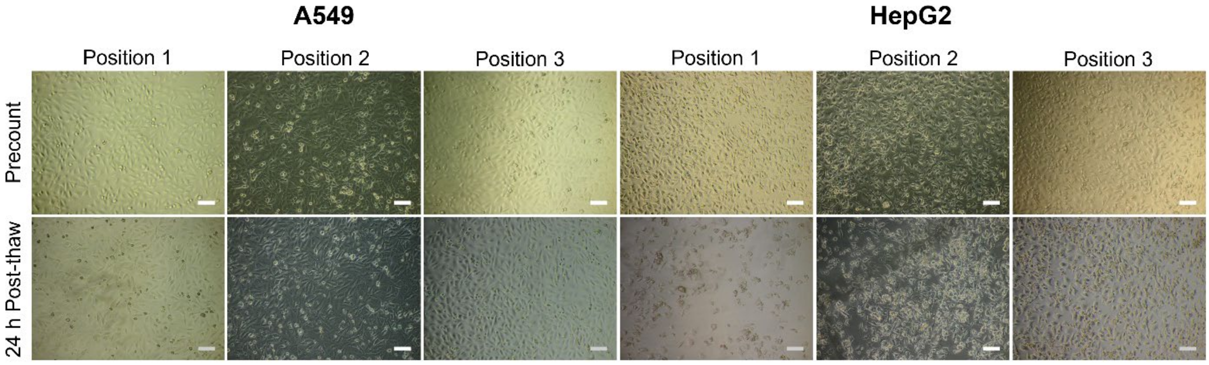

Figure S9. Post-thaw images of multiple positions within well plates. A549 (300k cell per well) and HepG2 (400k cell per well) cells were seeded as confluent monolayers, frozen with 10 %v/v DMSO and 40 mg.mL^{-1} of polyampholyte and imaged 24 h post-thaw at 3 different positions. Non-frozen (precount) images have also been provided. Scale bar = 100 µm.

1.4 Resazurin cell viability assay

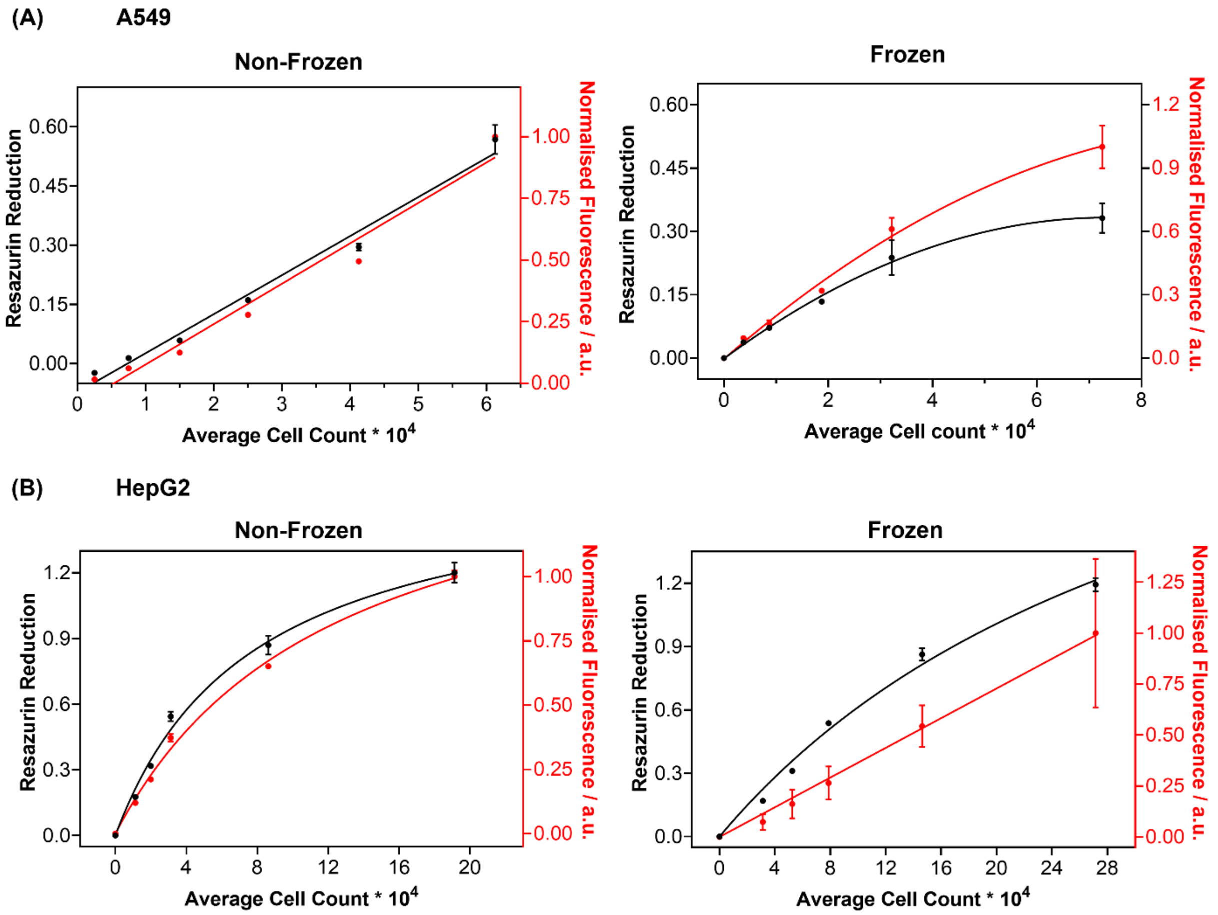

Figure S10. Resazurin reduction cell viability assay. Non-frozen and frozen (**A**) A549 and (**B**) HepG2 cells, seeded at multiple cell densities, were incubated with resazurin solution 24 h post-thaw and the conversion of resazurin to resorufin was measured by absorbance and fluorescence. Data is presented as both resazurin reduction and normalised fluorescence ± SEM from two independent repeats.

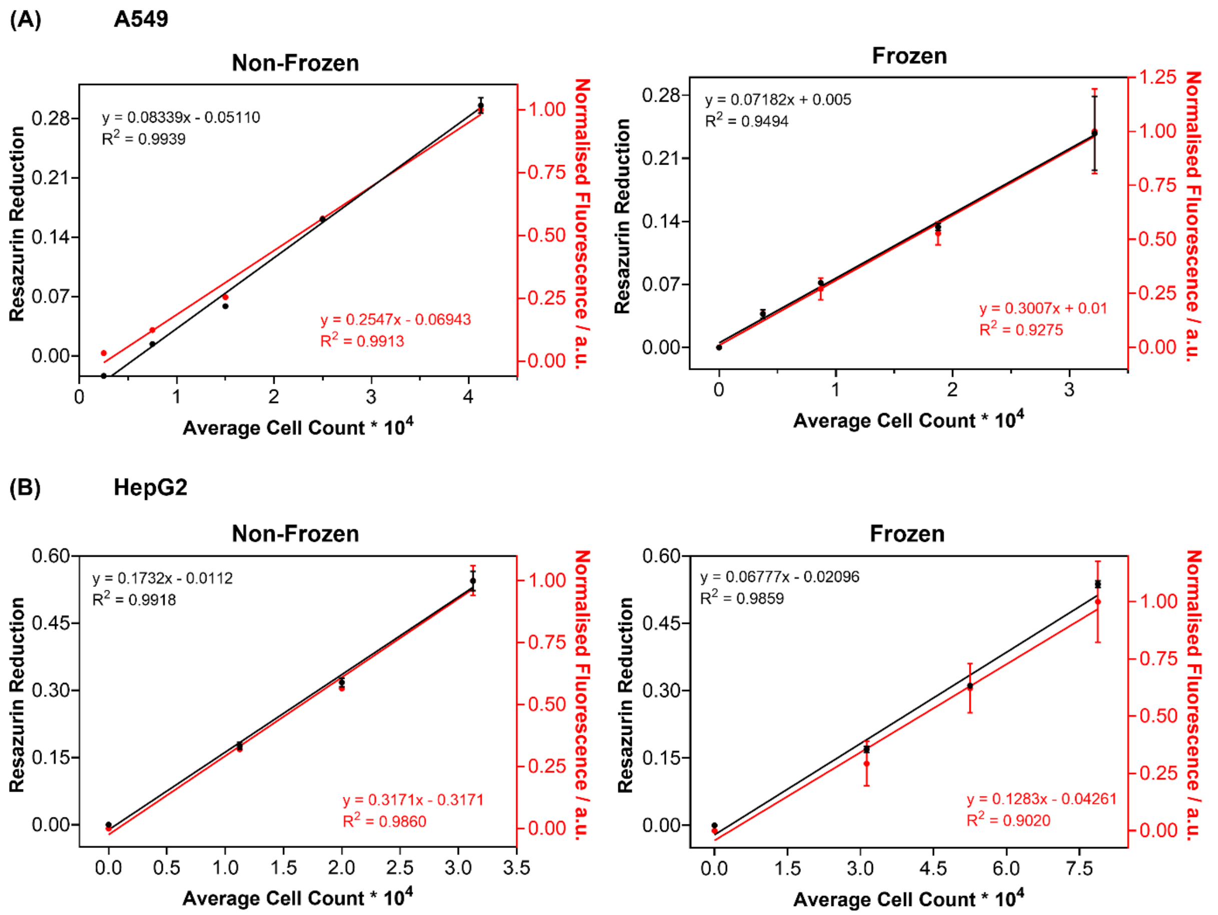

Figure S11. Linear dynamic working range of resazurin reduction assay. Graphs from Fig. S10 were replotted to illustrate the linear working range for % cell viability measurements in drug screening applications. Data is presented as both resazurin reduction and normalised fluorescence ± SEM from two independent repeats.

1.12 Imaging intracellular ice growth

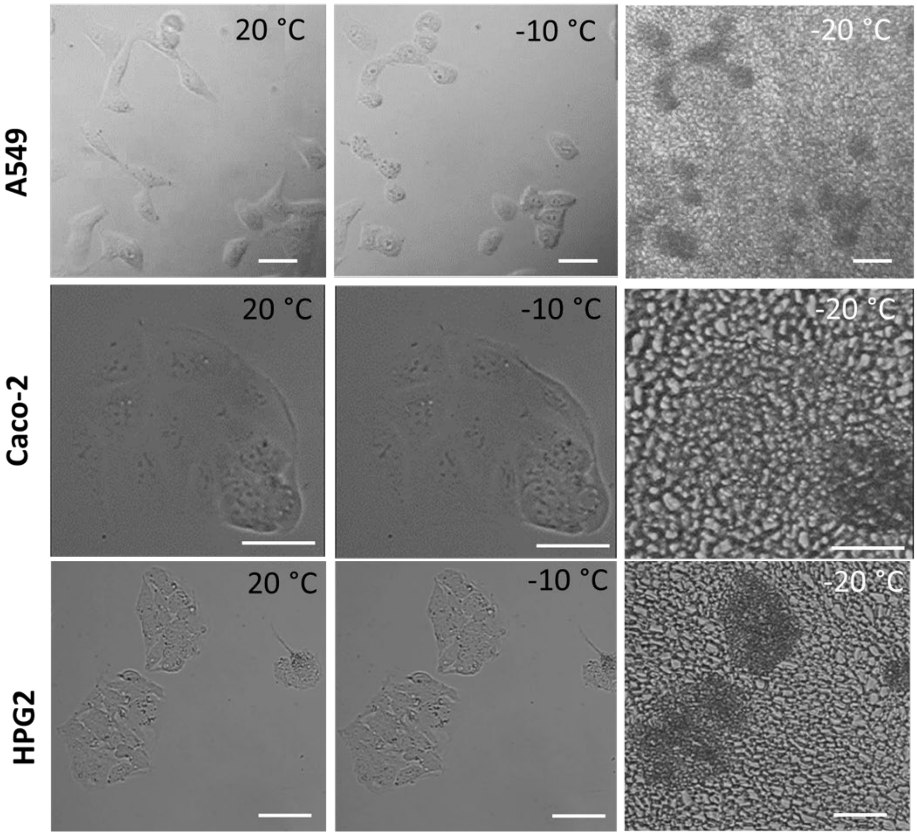

Figure S12. Cryomicroscopy images. A549, HepG2 and Caco-2 cells were frozen on a cryostage in the presence of 10 %v/v DMSO and 40 mg.mL^{-1} of polyampholyte to visualise intracellular ice growth. Images were taken at RT (20 °C), -10 °C and -20 °C. Scale bar = 100 μm.

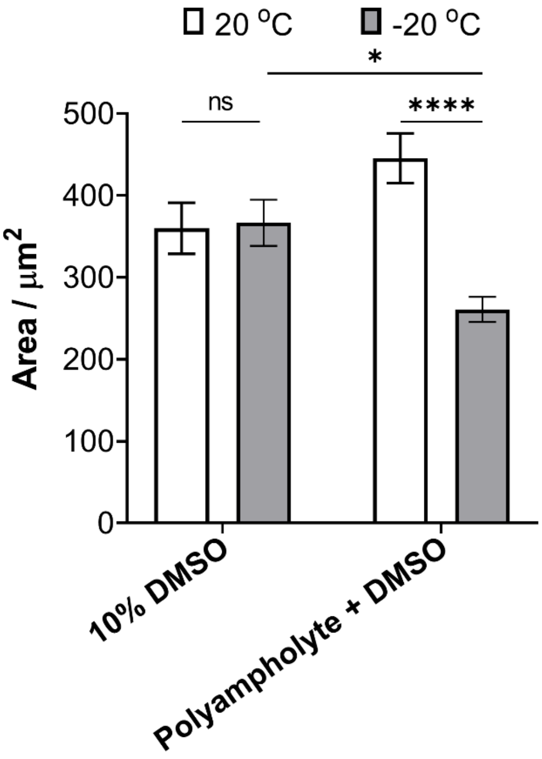

Figure S13. Cellular dehydration during freezing. A549 cells were imaged using cryomicroscopy at 20 °C and -20 °C in media containing either 10 %v/v DMSO (white bar) or 10 %v/v DMSO and 40 mg.mL^{-1} of polyampholyte (dark grey bar) to quantify cell shrinkage (by area) using ImageJ. The data is presented as mean area ± SEM of at least 37 cells imaged (ANOVA, Tukey PostHoc; ns: $p > 0.05$, * $p \leq 0.05$, **** $p \leq 0.0001$).

1.6 Caspase-3/-7 assay

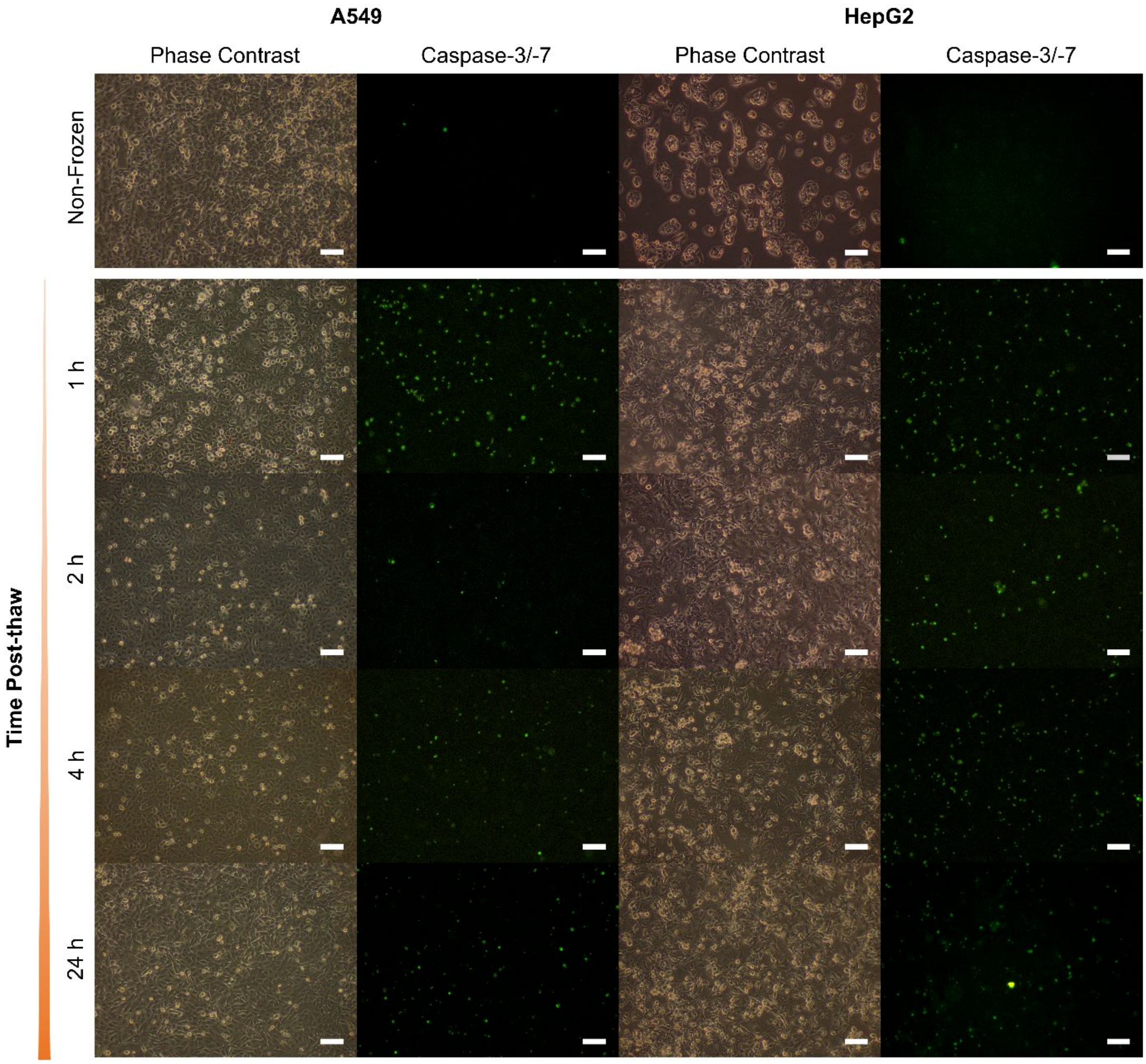

Figure S14. Imaging caspase-3/-7 activation. A549 and HepG2 cells frozen with 40 mg.mL^{-1} of polyampholyte and 10% DMSO were thawed with cell culture media and incubated with CellEvent Caspase-3/7 Detection Reagent for real-time imaging of caspase activation over time. Representative images have been provided.

1.9 Cell cycle analysis

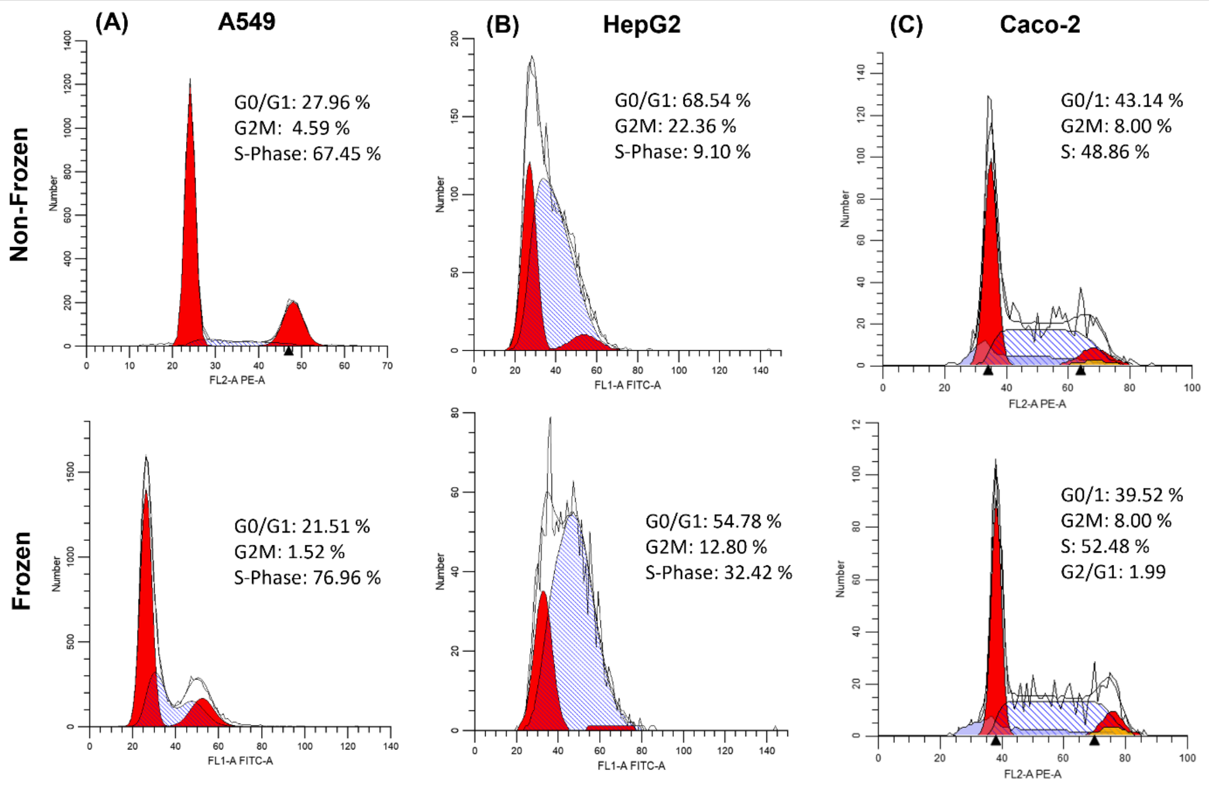

Figure S15. Flow cytometry cell cycle analysis of (**A**) A549, (**B**) HepG2 and (**C**) Caco-2 before and 24h after freezing with 10% DMSO and 40 mg.mL^{-1} of polyampholyte.

1.10 CYP response

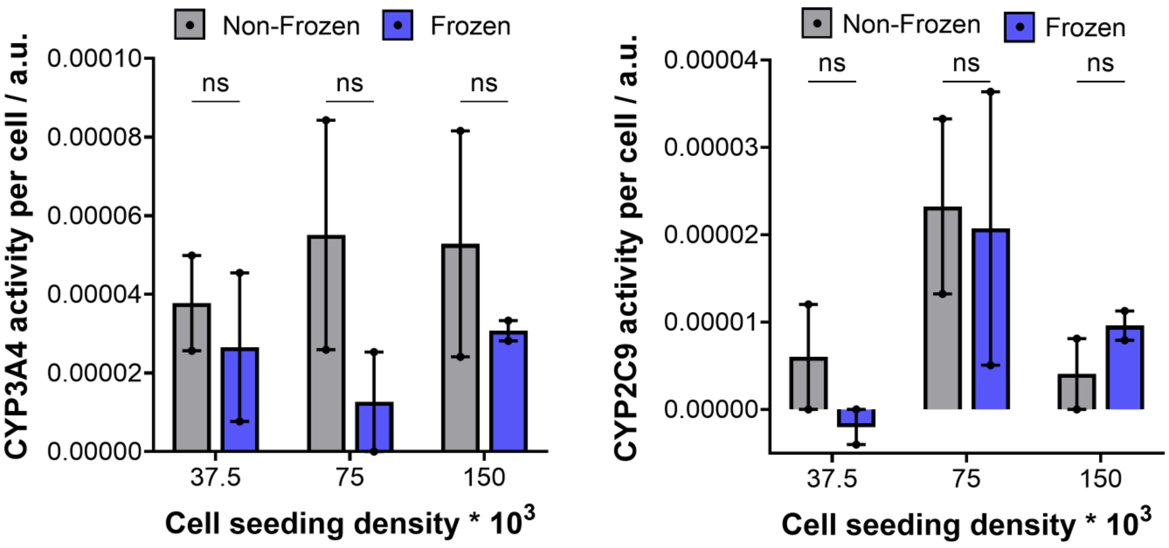

Figure S16. Innate cytochrome P450 (CYP) activity. The innate CYP activity of HepG2 cells was measured before and after freezing with 10% DMSO and 40 mg.mL^{-1} of polyampholyte. Data is presented as mean CYP activity per cell ± SEM from two independent repeats (ANOVA, Tukey PostHoc; ns: $p > 0.05$).

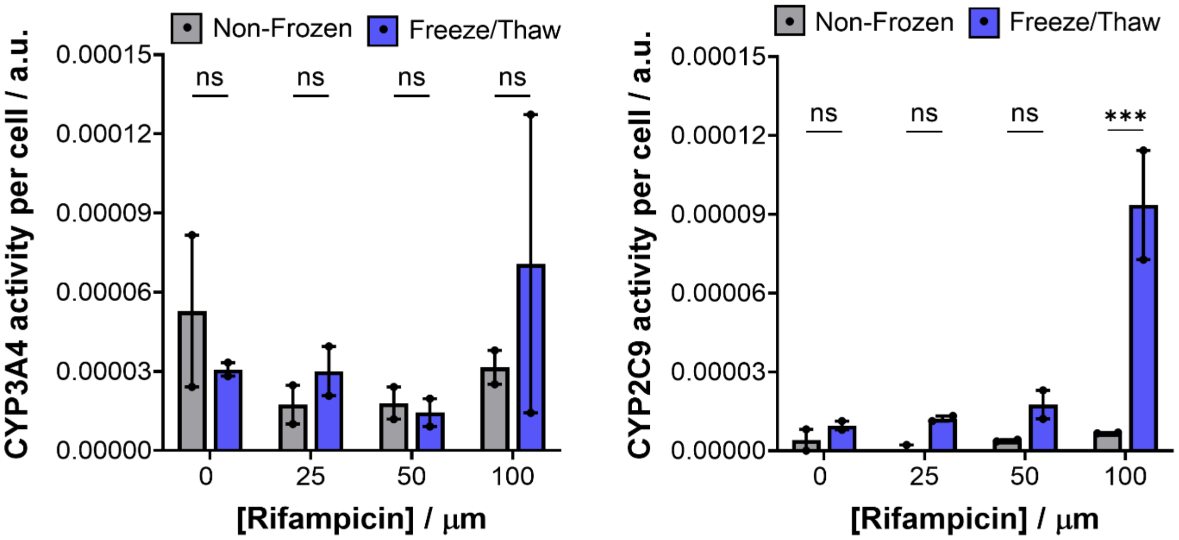

Figure S17. Rifampicin-induced cytochrome P450 (CYP) response. Cryopreserved confluent HepG2 cells, frozen with 10 %v/v DMSO and 40 mg.mL^{-1} of polyampholyte, were thawed for 24 h and incubated with Rifampicin for 48 h to elevate CYP responses. Data is presented as mean CYP activity per cell ± SEM from two independent repeats (ANOVA, Tukey PostHoc; ns: $p > 0.05$, *** $p \leq 0.001$).

1.11 Long term storage and transportation of frozen cells

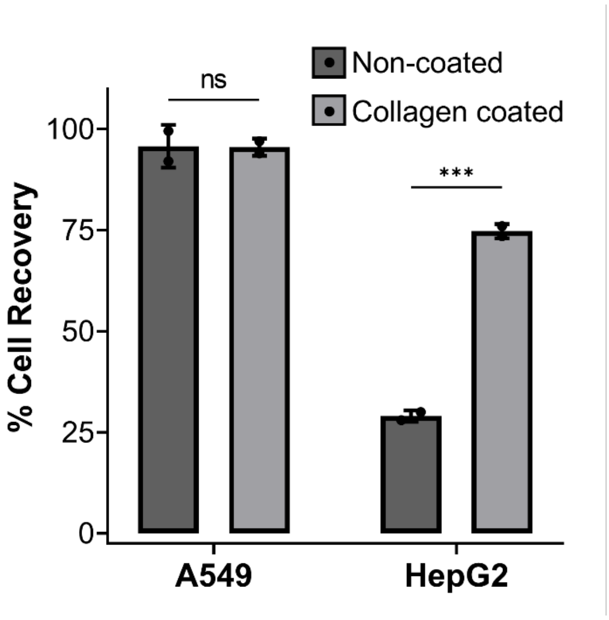

Figure S18. Storage in dry ice. A549 and HepG2 cells were frozen with 40 mg.mL^{-1} of polyampholyte and 10 %v/v DMSO in either collagen coated or uncoated plates. Plates were subsequently stored in polystyrene boxes containing dry ice for 5 days to determine feasibility of plates for transportation. The percentage of cells recovered were determined 24 h post-thaw and the data is presented as mean % cell recovery ± SEM from two independent repeats (ANOVA, Tukey PostHoc; ns: $p > 0.05$, *** $p \leq 0.001$).

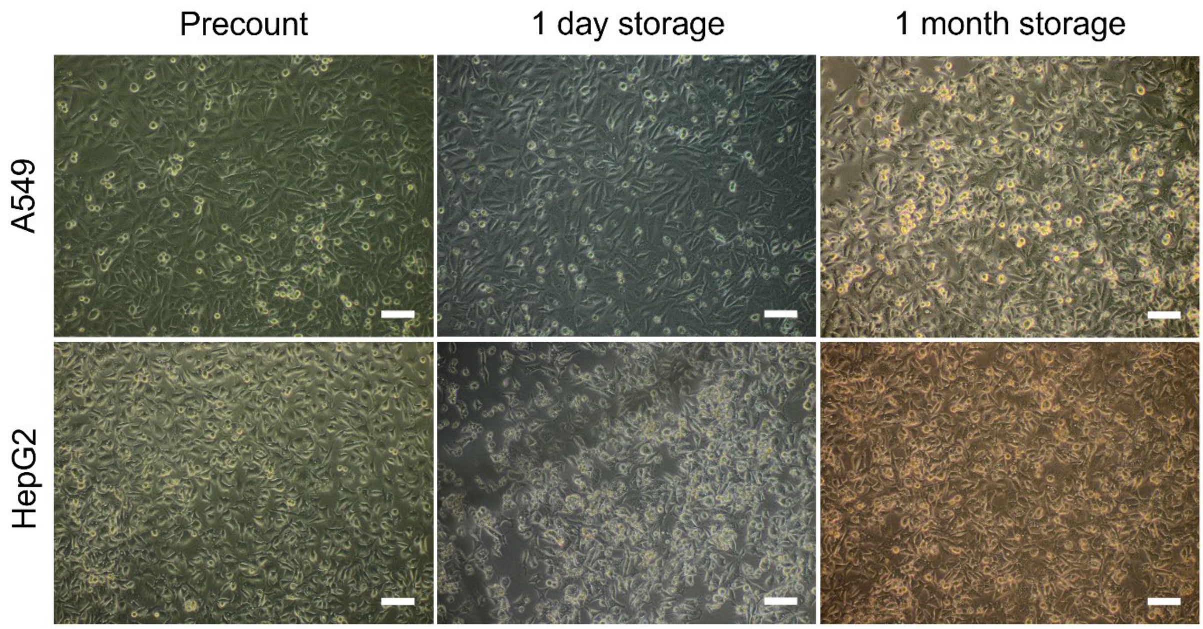

Figure S19. Long-term cell storage. A549 and HepG2 cells were imaged before freezing and 24 h post-thaw following storage for 1 day or 30 days in a -80 °C freezer. Scale bar = 100 µm.
